# Supplementary material for: “The impact of the COVID-19 pandemic on research activities: A survey of the largest Italian academic community”
Source: PLoS One. 2024 Jun 25;19(6):e0304078. doi: 10.1371/journal.pone.0304078 (PMC11198756; doi:10.1371/journal.pone.0304078)
Supplement: S1 Appendix — (DOCX) [file pone.0304078.s001.docx]

**Sapienza Ricerca: restart after the pandemic**

**Demographic information**

- Sex
- Age

**Academic position**

- Full Professor
- Associate Professor
- *”Ricercatore a tempo indeterminato”*
- *“Ricercatore a tempo determinato tipo B [RTDb]"*
- *“Ricercatore a tempo determinato tipo A [RTDa]"*
- *"Assegnista di ricerca"*
- Ph.D. student

**University affiliation**

- Faculty
- Department

**Describe your research activity**

- Theoretical Research
- Technical/Applied Research
- Biological Research
- Basic Research
- Clinical Research
- Field Research

**Main issue faced during the pandemic (select one)**

- Start of the experimentation / obtaining authorizations
- Progress of the experimentation
- Retrieval of sources
- Access to libraries / laboratories / research sites
- Patient recruitment / access
- Logistics linked to transport
- Family organization
- Expiry of contracts for personnel recruited specifically for research
- Expiry of consumables
- Economic reporting of projects
- Scientific production
- Access to grants / Funding
- Limitations to periods of stay / study / research abroad
- Limitations to the provision of services by third parties

**Additional issues (multiple responses allowed)**

- Start of the experimentation / obtaining authorizations
- Progress of the experimentation
- Retrieval of sources
- Access to libraries / laboratories / research sites
- Patient recruitment / access
- Logistics linked to transport
- Family organization
- Expiry of contracts for personnel recruited specifically for research
- Expiry of consumables
- Economic reporting of projects
- Scientific production
- Access to grants / Funding
- Limitations to periods of stay / study / research abroad
- Limitations to the provision of services by third parties

**Economic impact**

- No economic impact
- <10.000 €
- 10.000 - 30.000 €
- 30.000 - 60.000 €
- 60.000 - 100.000 €
- >100.000 €

**Motivate the economic impact (open question)**

**Suggestions and proposals for the relaunch of research activities after the pandemic (open question)**
