# Supplementary material for: “The impact of the COVID-19 pandemic on research activities: A survey of the largest Italian academic community”
Source: PLoS One. 2024 Jun 25;19(6):e0304078. doi: 10.1371/journal.pone.0304078 (PMC11198756; doi:10.1371/journal.pone.0304078)
Supplement: Attachment — Submitted filename: Response to Reviewer .docx [file pone.0304078.s003.docx]

Journal requirements:

When submitting your revision, we need you to address these additional requirements.

1. Please ensure that your manuscript meets PLOS ONE's style requirements, including those for file naming. The PLOS ONE style templates can be found at

<https://journals.plos.org/plosone/s/file?id=wjVg/PLOSOne_formatting_sample_main_body.pdf> and

<https://journals.plos.org/plosone/s/file?id=ba62/PLOSOne_formatting_sample_title_authors_affiliations.pdf>

Answer: done

2. Please provide additional details regarding participant consent. In the ethics statement in the Methods and online submission information, please ensure that you have specified (1) whether consent was informed and (2) what type you obtained (for instance, written or verbal, and if verbal, how it was documented and witnessed). If your study included minors, state whether you obtained consent from parents or guardians. If the need for consent was waived by the ethics committee, please include this information.

If you are reporting a retrospective study of medical records or archived samples, please ensure that you have discussed whether all data were fully anonymized before you accessed them and/or whether the IRB or ethics committee waived the requirement for informed consent. If patients provided informed written consent to have data from their medical records used in research, please include this information

Once you have amended this/these statement(s) in the Methods section of the manuscript, please add the same text to the “Ethics Statement” field of the submission form (via “Edit Submission”).

For additional information about PLOS ONE ethical requirements for human subjects research, please refer to <http://journals.plos.org/plosone/s/submission-guidelines#loc-human-subjects-research>.

Response: done

3. We note that you have included the phrase “data not shown” in your manuscript. Unfortunately, this does not meet our data sharing requirements. PLOS does not permit references to inaccessible data. We require that authors provide all relevant data within the paper, Supporting Information files, or in an acceptable, public repository. Please add a citation to support this phrase or upload the data that corresponds with these findings to a stable repository (such as Figshare or Dryad) and provide and URLs, DOIs, or accession numbers that may be used to access these data. Or, if the data are not a core part of the research being presented in your study, we ask that you remove the phrase that refers to these data.

Response: Following journal’s requirements, we have uploaded the full anonymized dataset underlying the findings described in this manuscript in Figshare. DOI to uploaded file is:

10.6084/m9.figshare.23551743 We have integrated the materials and methods section specifying that the full dataset has been uploaded in Figshare. Data Availability Statement: Data is available from Figshare (DOI: 10.6084/m9.figshare.23551743).

4. Check the last line of the abstract to ensure it is the same.  The reason for this check is to ensure that the AEs and Reviewers are sent correct information to allow them to make a good decision on whether they can manage/review the manuscript.  Only send back for a change if the abstract on EM and in the manuscript are VASTLY different.

Response: Dear Editor, the abstract has been changed.

[Note: HTML markup is below. Please do not edit.]

Reviewers' comments:

Reviewer's Responses to Questions

**Comments to the Author**

1. Is the manuscript technically sound, and do the data support the conclusions?

The manuscript must describe a technically sound piece of scientific research with data that supports the conclusions. Experiments must have been conducted rigorously, with appropriate controls, replication, and sample sizes. The conclusions must be drawn appropriately based on the data presented.

Reviewer #1: Partly

Reviewer #2: Partly

2. Has the statistical analysis been performed appropriately and rigorously?

Reviewer #1: Yes

Reviewer #2: No

3. Have the authors made all data underlying the findings in their manuscript fully available?

The [PLOS Data policy](http://www.plosone.org/static/policies.action#sharing) requires authors to make all data underlying the findings described in their manuscript fully available without restriction, with rare exception (please refer to the Data Availability Statement in the manuscript PDF file). The data should be provided as part of the manuscript or its supporting information, or deposited to a public repository. For example, in addition to summary statistics, the data points behind means, medians and variance measures should be available. If there are restrictions on publicly sharing data—e.g. participant privacy or use of data from a third party—those must be specified.

Reviewer #1: No

Reviewer #2: No

4. Is the manuscript presented in an intelligible fashion and written in standard English?

PLOS ONE does not copyedit accepted manuscripts, so the language in submitted articles must be clear, correct, and unambiguous. Any typographical or grammatical errors should be corrected at revision, so please note any specific errors here.

Reviewer #1: No

Reviewer #2: No

5. Review Comments to the Author

Please use the space provided to explain your answers to the questions above. You may also include additional comments for the author, including concerns about dual publication, research ethics, or publication ethics. (Please upload your review as an attachment if it exceeds 20,000 characters)

Reviewer #1: Abstract;
Needs some review
Introduction:

The authors proposed an interesting research, however the introduction needs to some adjustments. What is the context of the study (needs improvement); explore the importance of the theme (needs improvement); the main goals and relevance of the study (good) how this study will contribute (add).

Response: We thank the reviewer for this comment. We have largely modified the introduction. In particular, we added in the introduction section paragraphs giving more insights and context in terms of existing literature, the importance of the subject matter, and the relative contribution of our study to the scientific knowledge that is currently available. Please see the red parts in the text.

L60-61 discuss better: how double/triple journey of female researcher would be impacted specific in the context of the pandemic? The sentence is very general and accounts for what is already known. Ref.: Davis, P.B., Meagher, E.A., Pomeroy, C. et al. Pandemic-related barriers to the success of women in research: a framework for action. Nat Med 28, 436–438 (2022). <https://doi.org/10.1038/s41591-022-01692-8>

Response: We thank the reviewer for this comment. Gender-related issues in research and teaching during the pandemic have been largely addressed in the new version with the relevant references. Generally, it seems that pre-existing barriers to academic activity have become amplified during the COVID-19 pandemic. Our results are not fully consistent with some of the previous reports, probably as a consequence of some features of the information required in the questionnaires which we acknowledged as a limitation. We have therefore added a paragraph about female researchers facing the pandemic in the introduction as well as a specific discussion. Please see red parts in the text.

L62-64 what about the race to research and publish articles in health science? Investments in vaccines? Data sharing and cooperation internationally?

Response: We thank the reviewer for this comment. This is a complex issue. During the pandemic, research activities related to Covid-19 have been privileged with a consequent increase in publication of COVID-19 related papers also owing to international unpreceded data sharing. Despite this, a reduction in international cooperation and and meetings in other fields has been observed and the accuracy of fast-track COVID-19 related publications has also been questioned. We have added multiple sentences in both the introduction and discussion about this. Please see red text in the manuscript (see introduction line 71-75; discussion page 26). This aspect was not an objective of our survey although it has been extensively investigated in previous studies as we mentioned in the introduction and discussion.

Methods
Why you don't have ethical approval for this research?
How researchers developed the informed consent for participants? how you developed the questionnaires? you should at least get a weaver from the ethical committee of the university. Please contact the ethical committee of your institution and clarify this.

Response: please see method section for the ethical approval.

Results

Table 1
research type - check "filed"is it a research type? I don't understand.

Response: We are afraid the Reviewer may have misread “field (research)” as “filed (research)”. We intend Field research (or study) to comprise all subjects in which raw data is collected outside of a laboratory, workplace or library setting. This large field comprises social sciences observing people interact in their natural environment, biologists studying natural animal behaviour, and many archaeologists, anthropologists, economists, etc.

Supplementary data
make sure to add data that are relevant to the study. Check the journal policy regarding data sharing and transparency in order to be in accordance to it

Response: We thank the Reviewer for his/her correct observation. We now provide the complete anonymized Excel database used for the current research and all the statistical analyses.

Conclusions

Make sure to add a bullet point

Response: We thank the reviewer for this comment. We don’t know if a bullet point it is appropriate as for journal style. If so, we have provided a separate file with the bullet list.

Limitations

It is always interesting to point it out

Response: We thank the Reviewer for this comment. We added a Limitations section.

Reviewer #2: The authors attempted to assess the effects of the COVID-19 pandemic on research activities of a univeristy in Italy and identify the most critical issues, from a cross-sectional study. The findings may provide some evidence of the pandemic impacts on the research community in an insitution.

The manuscript requires some substantial revisions before consideration of the journal:
1. the literature review was not organised and presented with sufficient evidence

Response: we thank this reviewer for the comment. We have largely integrated both introduction and discussion with the available pertinent literature. Please see red text.

2. the statistical analysis part was not elaborated and explained clealry, especially how the criteria and assumptions of a partciular statistical analysis were met, e.g. some questions in the survey allow selecting multiple answers, how did these considered in the statistical tests

R. We thank Reviewer #2 for his considerations and constructive criticism. Survey questions that allowed multiple answers were analysed and presented as follows: 1) in Table 2 with regards to the description of the research-related issues (no statistical testing); 2) in Tables 3 through 4 with regards to the overall research-related issues experienced based on academic position and according to the impact of gender, age, academic field (using ANOVAs, with no post-hoc testing); 3) in Tables 5 and 8 to assess independent predictors of the overall number of research related issues and the presence of economic burden (using multiple linear regression); 4) in Tables 6 and 7 to describe and assess the reasons for economic burden and suggestions to improve academic research, according to above-mentioned subgroups (using ANOVAs, with no post-hoc testing).

Given the exploratory nature of our study, in which data are collected with an objective (*“to explore the impact of the COVID-19 pandemic on research activities and in the attempt to identify the most critical issues”*), rather than with prespecified key hypotheses, we chose not to adjust for multiple comparisons, according to relevant literature on the topic ([10.1530/EJE-20-1375](https://doi.org/10.1530/EJE-20-1375), [10.1016/j.joca.2016.01.008](https://doi.org/10.1016/j.joca.2016.01.008), [10.1016/S0895-4356(00)00314-0](https://doi.org/10.1016/S0895-4356(00)00314-0)). As such we place low emphasis on specific significant comparisons, but rather focus on the description of the research cohort and on exploring risk factors for greater research burden. Lastly, we employed bootstrapping on 2000 samples as a robust regression methods to account for non-normal data distribution, reporting bias-corrected accelerated (BCa) 95% confidence intervals and adjusted R^2^ values (according to Nagelkerke) to describe the fit of our models. We have made these points clearer to the reader in the manuscript.

3. the survey adopted was self-designed, some elaboration of the evidence of referencing to literature is required

Response: we thank the Reviewer for his/her correct observation. The questionnaire was indeed self-designed from the authors, who serve(d) as Governance Research Team at Sapienza University of Rome, based on the available literature on the topic, referenced in the Introduction and the Discussion sections. Specifically, the development of the survey focused on exploring the differential impact of the pandemic on different academic subgroups, expecting a greater toll on women (PMID 32409466), younger researchers and Ph.D. students (PMID **32493949),** specific research activities (PMID **36530543**), and researchers involved in clinical trials (35050229), etc. All these aspects are now better discussed in the Introduction section and the methodology is more clearly presented in Lines 149-151.

4. the discussion part appears lacking in-depth discussion, some substantiation of the literature is required

Response: we thank this reviewer for the comment. We have largely integrated both introduction and discussion with available literature. Please see red text.

5. an Italian academic institution was selected as the study site, given its specificity in its organization/system/roles of academic personnel as stated by the authors, the authors are required to add to explain how the findings would be useful to the wider academic community in other countries

Response: we thank this reviewer for the comment. As now clearly stated in the text, Sapienza is the largest university in Italy with many excellent departments. Academics of all degrees have taken part to this survey, therefore the authors think that results could be useful to a wider academic community. In order to meet this appropriate remark of the reviewer, we also proposed a modified title of the article which reflects the impact of pandemic of a very representative academic research community and on potential other institutions.
