# Supplementary material for: “The impact of the COVID-19 pandemic on research activities: A survey of the largest Italian academic community”
Source: PLoS One. 2024 Jun 25;19(6):e0304078. doi: 10.1371/journal.pone.0304078 (PMC11198756; doi:10.1371/journal.pone.0304078)
Supplement: Attachment — Submitted filename: PONE-D-22-11862_R1.docx [file pone.0304078.s004.docx]

PLOS ONE

“The impact of the COVID-19 pandemic on research activities in the largest Italian academic community”

--Manuscript Draft--

| Manuscript Number: | PONE-D-22-11862R1 |
| --- | --- |
| Article Type: | Research Article |
| Full Title: | “The impact of the COVID-19 pandemic on research activities in the largest Italian academic community” |
| Short Title: | HOW PANDEMIC HAS CHANGED THE WORLD OF ACADEMIC RESEARCH |
| Corresponding Author: | Massimo Volpe, M,D.  Sapienza University of Rome: Universita degli Studi di Roma La Sapienza Rome, ITALY |
| Keywords: | COVID-19; pandemic; academic community; academic research |
| Abstract: | Purpose: The aim of the present work is to explore the impact of the COVID-19 pandemic on research activities in a vast multidisciplinary academic community in order to identify the most critical issues.  Method: To this purpose we planned a survey addressed to the entire academic research staff at "Sapienza" University of Rome, which represents the largest Italian academic community. A questionnaire consisting of both open and closed questions was delivered to 4118 individuals in April 2021.  Results: 544 responses were collected. All academic roles were sufficiently represented in the research cohort. The median number of critical issues experienced by academic research staff was three. Among these, the three most frequently reported were related to: "Access to libraries / laboratories / research sites" (21.9%),  "Limitation to stay abroad / study / research periods" (17.6%), "Progress of experimental work" (14.7%), with variable prevalence according to academic position and gender. Older subjects reported issues with "Projects’ financial reporting” and “Expiration of acquired consumable material more frequently”. The most common critical aspects reported in relation to the economic burden were: being “Unable to allocate funds” (31.4%), a “Reduction in clinical and scientific activity” (26.3%) and experiencing “Increased expenses (comprising private costs)” (21.2%) with no differences among genders. Researchers in Applied Sciences and Natural Sciences reported a higher frequency of problems in clinical and scientific activities, whereas increased expenses were reported also by researchers operating in the Humanities field. As a possible solution aimed at improving these critical issues, most subjects, especially those aged >45 years, indicated “Economic aid” (22.6%), “Reduction in bureaucracy” (19.9%) or “Enhancement of the scientific and clinical activities”, whereas those ≤45 years felt that an increased duration of and better access to PhD programmes were to be prioritized.  Conclusion: Our findings highlight the most critical issues related to research activities during COVID-19 pandemic in a large academic community. The information achieved may be useful to identify researchers’ needs and to design appropriate policies aimed at preparing research institutions for unexpected catastrophic events and limiting the negative impact on academic research activities. |
| Order of Authors: | Massimo Volpe, M,D. |
|  | Massimo Ralli |
|  | Andrea Isidori |
| Response to Reviewers: | Journal requirements:  When submitting your revision, we need you to address these additional requirements.  1. Please ensure that your manuscript meets PLOS ONE's style requirements, including those for file naming. The PLOS ONE style templates can be found at https://journals.plos.org/plosone/s/file?id=wjVg/PLOSOne_formatting_sample_main_bo dy.pdf and  https://journals.plos.org/plosone/s/file?id=ba62/PLOSOne_formatting_sample_title_aut hors_affiliations.pdf |

|  | Answer: done  2. Please provide additional details regarding participant consent. In the ethics statement in the Methods and online submission information, please ensure that you have specified (1) whether consent was informed and (2) what type you obtained (for instance, written or verbal, and if verbal, how it was documented and witnessed). If your study included minors, state whether you obtained consent from parents or guardians. If the need for consent was waived by the ethics committee, please include this information.  If you are reporting a retrospective study of medical records or archived samples, please ensure that you have discussed whether all data were fully anonymized before you accessed them and/or whether the IRB or ethics committee waived the  requirement for informed consent. If patients provided informed written consent to have data from their medical records used in research, please include this information Once you have amended this/these statement(s) in the Methods section of the manuscript, please add the same text to the “Ethics Statement” field of the submission form (via “Edit Submission”).  For additional information about PLOS ONE ethical requirements for human subjects research, please refer to http://journals.plos.org/plosone/s/submission-guidelines#lochuman-subjects-research.  Response: done   3. We note that you have included the phrase “data not shown” in your manuscript.Unfortunately, this does not meet our data sharing requirements. PLOS does not permit references to inaccessible data. We require that authors provide all relevant data within the paper, Supporting Information files, or in an acceptable, public repository. Please add a citation to support this phrase or upload the data that corresponds with these findings to a stable repository (such as Figshare or Dryad) and provide and URLs, DOIs, or accession numbers that may be used to access these data. Or, if the data are not a core part of the research being presented in your study, we ask that you remove the phrase that refers to these data.   Response: Following journal’s requirements, we have uploaded the full anonymized dataset underlying the findings described in this manuscript in Figshare. DOI to uploaded file is:  10.6084/m9.figshare.23551743 We have integrated the materials and methods section specifying that the full dataset has been uploaded in Figshare. Data Availability Statement: Data is available from Figshare (DOI: 10.6084/m9.figshare.23551743).   4. Check the last line of the abstract to ensure it is the same. The reason for this checkis to ensure that the AEs and Reviewers are sent correct information to allow them to make a good decision on whether they can manage/review the manuscript. Only send back for a change if the abstract on EM and in the manuscript are VASTLY different. Response: Dear Editor, the abstract has been changed.   [Note: HTML markup is below. Please do not edit.] Reviewers' comments:  Reviewer's Responses to Questions Comments to the Author   1. Is the manuscript technically sound, and do the data support the conclusions?   The manuscript must describe a technically sound piece of scientific research with data that supports the conclusions. Experiments must have been conducted rigorously, with appropriate controls, replication, and sample sizes. The conclusions must be drawn appropriately based on the data presented.  Reviewer #1: Partly Reviewer #2: Partly   2. Has the statistical analysis been performed appropriately and rigorously? Reviewer #1: Yes Reviewer #2: No 3. Have the authors made all data underlying the findings in their manuscript fullyavailable? |
| --- | --- |

|  | The PLOS Data policy requires authors to make all data underlying the findings described in their manuscript fully available without restriction, with rare exception (please refer to the Data Availability Statement in the manuscript PDF file). The data should be provided as part of the manuscript or its supporting information, or deposited to a public repository. For example, in addition to summary statistics, the data points behind means, medians and variance measures should be available. If there are restrictions on publicly sharing data—e.g. participant privacy or use of data from a third party—those must be specified.  Reviewer #1: No Reviewer #2: No   4. Is the manuscript presented in an intelligible fashion and written in standardEnglish?   PLOS ONE does not copyedit accepted manuscripts, so the language in submitted articles must be clear, correct, and unambiguous. Any typographical or grammatical errors should be corrected at revision, so please note any specific errors here. Reviewer #1: No Reviewer #2: No   5. Review Comments to the Author   Please use the space provided to explain your answers to the questions above. You may also include additional comments for the author, including concerns about dual publication, research ethics, or publication ethics. (Please upload your review as an attachment if it exceeds 20,000 characters)  Reviewer #1: Abstract;  Needs some review Introduction:  The authors proposed an interesting research, however the introduction needs to some adjustments. What is the context of the study (needs improvement); explore the importance of the theme (needs improvement); the main goals and relevance of the study (good) how this study will contribute (add).  Response: We thank the reviewer for this comment. We have largely modified the introduction. In particular, we added in the introduction section paragraphs giving more insights and context in terms of existing literature, the importance of the subject matter, and the relative contribution of our study to the scientific knowledge that is currently available. Please see the red parts in the text.  L60-61 discuss better: how double/triple journey of female researcher would be impacted specific in the context of the pandemic? The sentence is very general and accounts for what is already known. Ref.: Davis, P.B., Meagher, E.A., Pomeroy, C. et al. Pandemic-related barriers to the success of women in research: a framework for action. Nat Med 28, 436–438 (2022). https://doi.org/10.1038/s41591-022-01692-8 Response: We thank the reviewer for this comment. Gender-related issues in research and teaching during the pandemic have been largely addressed in the new version with the relevant references. Generally, it seems that pre-existing barriers to academic activity have become amplified during the COVID-19 pandemic. Our results are not fully consistent with some of the previous reports, probably as a consequence of some features of the information required in the questionnaires which we acknowledged as a limitation. We have therefore added a paragraph about female researchers facing the pandemic in the introduction as well as a specific discussion. Please see red parts in the text.  L62-64 what about the race to research and publish articles in health science?  Investments in vaccines? Data sharing and cooperation internationally?  Response: We thank the reviewer for this comment. This is a complex issue. During the pandemic, research activities related to Covid-19 have been privileged with a consequent increase in publication of COVID-19 related papers also owing to international unpreceded data sharing. Despite this, a reduction in international cooperation and and meetings in other fields has been observed and the accuracy of fast-track COVID-19 related publications has also been questioned. We have added multiple sentences in both the introduction and discussion about this. Please see red |
| --- | --- |

|  | text in the manuscript (see introduction line 71-75; discussion page 26). This aspect was not an objective of our survey although it has been extensively investigated in previous studies as we mentioned in the introduction and discussion.  Methods  Why you don't have ethical approval for this research?  How researchers developed the informed consent for participants? how you developed the questionnaires? you should at least get a weaver from the ethical committee of the university. Please contact the ethical committee of your institution and clarify this. Response: please see method section for the ethical approval.  Results  Table 1 research type - check "filed"is it a research type? I don't understand.  Response: We are afraid the Reviewer may have misread “field (research)” as “filed (research)”. We intend Field research (or study) to comprise all subjects in which raw data is collected outside of a laboratory, workplace or library setting. This large field comprises social sciences observing people interact in their natural environment, biologists studying natural animal behaviour, and many archaeologists, anthropologists, economists, etc.  Supplementary data  make sure to add data that are relevant to the study. Check the journal policy regarding data sharing and transparency in order to be in accordance to it  Response: We thank the Reviewer for his/her correct observation. We now provide the complete anonymized Excel database used for the current research and all the statistical analyses.  Conclusions  Make sure to add a bullet point  Response: We thank the reviewer for this comment. We don’t know if a bullet point it is appropriate as for journal style. If so, we have provided a separate file with the bullet list.  Limitations  It is always interesting to point it out  Response: We thank the Reviewer for this comment. We added a Limitations section.  Reviewer #2: The authors attempted to assess the effects of the COVID-19 pandemic on research activities of a univeristy in Italy and identify the most critical issues, from a cross-sectional study. The findings may provide some evidence of the pandemic impacts on the research community in an insitution.  The manuscript requires some substantial revisions before consideration of the journal:   1. the literature review was not organised and presented with sufficient evidenceResponse: we thank this reviewer for the comment. We have largely integrated both introduction and discussion with the available pertinent literature. Please see red text. 2. the statistical analysis part was not elaborated and explained clealry, especially howthe criteria and assumptions of a partciular statistical analysis were met, e.g. some questions in the survey allow selecting multiple answers, how did these considered in the statistical tests   R. We thank Reviewer #2 for his considerations and constructive criticism. Survey questions that allowed multiple answers were analysed and presented as follows: 1) in Table 2 with regards to the description of the research-related issues (no statistical testing); 2) in Tables 3 through 4 with regards to the overall research-related issues experienced based on academic position and according to the impact of gender, age, academic field (using ANOVAs, with no post-hoc testing); 3) in Tables 5 and 8 to assess independent predictors of the overall number of research related issues and the presence of economic burden (using multiple linear regression); 4) in Tables 6 and 7 to describe and assess the reasons for economic burden and suggestions to improve |
| --- | --- |

|  | academic research, according to above-mentioned subgroups (using ANOVAs, with no post-hoc testing).  Given the exploratory nature of our study, in which data are collected with an objective (“to explore the impact of the COVID-19 pandemic on research activities and in the attempt to identify the most critical issues”), rather than with prespecified key hypotheses, we chose not to adjust for multiple comparisons, according to relevant literature on the topic (10.1530/EJE-20-1375, 10.1016/j.joca.2016.01.008,  10.1016/S0895-4356(00)00314-0). As such we place low emphasis on specific significant comparisons, but rather focus on the description of the research cohort and on exploring risk factors for greater research burden. Lastly, we employed bootstrapping on 2000 samples as a robust regression methods to account for nonnormal data distribution, reporting bias-corrected accelerated (BCa) 95% confidence intervals and adjusted R2 values (according to Nagelkerke) to describe the fit of our models. We have made these points clearer to the reader in the manuscript.   3. the survey adopted was self-designed, some elaboration of the evidence ofreferencing to literature is required   Response: we thank the Reviewer for his/her correct observation. The questionnaire was indeed self-designed from the authors, who serve(d) as Governance Research Team at Sapienza University of Rome, based on the available literature on the topic, referenced in the Introduction and the Discussion sections. Specifically, the  development of the survey focused on exploring the differential impact of the pandemic on different academic subgroups, expecting a greater toll on women (PMID 32409466), younger researchers and Ph.D. students (PMID 32493949), specific research activities (PMID 36530543), and researchers involved in clinical trials (35050229), etc. All these aspects are now better discussed in the Introduction section and the methodology is more clearly presented in Lines 149-151.   4. the discussion part appears lacking in-depth discussion, some substantiation of theliterature is required   Response: we thank this reviewer for the comment. We have largely integrated both introduction and discussion with available literature. Please see red text.   5. an Italian academic institution was selected as the study site, given its specificity inits organization/system/roles of academic personnel as stated by the authors, the authors are required to add to explain how the findings would be useful to the wider academic community in other countries   Response: we thank this reviewer for the comment. As now clearly stated in the text, Sapienza is the largest university in Italy with many excellent departments. Academics of all degrees have taken part to this survey, therefore the authors think that results could be useful to a wider academic community. In order to meet this appropriate remark of the reviewer, we also proposed a modified title of the article which reflects the impact of pandemic of a very representative academic research community and on potential other institutions. |
| --- | --- |
| Additional Information: |  |
| Question | Response |
| Financial Disclosure  Enter a financial disclosure statement that describes the sources of funding for the work included in this submission. Review the [submission guidelines](http://journals.plos.org/plosone/s/submission-guidelines#loc-financial-disclosure-statement) for detailed requirements. View published research articles from [PLOS ONE](http://journals.plos.org/plosone/) for specific examples.  This statement is required for submission and will appear in the published article if | The author(s) received no specific funding for this work. |

| the submission is accepted. Please make sure it is accurate. |  |
| --- | --- |
| Unfunded studies  Enter: The author(s) received no specific funding for this work.  Funded studies  Enter a statement with the following details:   - Initials of the authors who received each award - Grant numbers awarded to each author - The full name of each funder - URL of each funder website - Did the sponsors or funders play any role in the study design, data collection and analysis, decision to publish, or preparation of the manuscript? - NO - Include this sentence at the end of your statement: The funders had no role in   study design, data collection and analysis, decision to publish, or preparation of the manuscript.   - YES - Specify the role(s) played. |  |
| * typeset |  |
| Competing Interests  Use the instructions below to enter a competing interest statement for this submission. On behalf of all authors, disclose any [competing interests](http://journals.plos.org/plosone/s/competing-interests) that could be perceived to bias this work—acknowledging all financial support and any other relevant financial or nonfinancial competing interests.  This statement is required for submission and will appear in the published article if the submission is accepted. Please make sure it is accurate and that any funding sources listed in your Funding Information later in the submission form are also declared in your Financial Disclosure statement.  View published research articles from [PLOS ONE](http://journals.plos.org/plosone/) for specific examples. | The authors have declared that no competing interests exist. |

| NO authors have competing interests  Enter: The authors have declared that no competing interests exist.  Authors with competing interests  Enter competing interest details beginning with this statement:  I have read the journal's policy and the authors of this manuscript have the following competing interests: [insert competing interests here] |  |
| --- | --- |
| * typeset |  |
| Ethics Statement  Enter an ethics statement for this submission. This statement is required if the study involved:   - Human participants - Human specimens or tissue - Vertebrate animals or cephalopods - Vertebrate embryos or tissues - Field research   Write "N/A" if the submission does not require an ethics statement.  General guidance is provided below. Consult the [submission guidelines](http://journals.plos.org/plosone/s/submission-guidelines#loc-guidelines-for-specific-study-types) for detailed instructions. Make sure that all information entered here is included in the Methods section of the manuscript. | The protocol for the current survey has been approved by the Sapienza Ethics Committee for Transdisciplinary Research (CERT) (approval no. CERT protocol ID 64/2023). |

| Format for specific study types  Human Subject Research (involving human participants and/or tissue)   - Give the name of the institutional review board or ethics committee that approved the study - Include the approval number and/or a statement indicating approval of this research - Indicate the form of consent obtained (written/oral) or the reason that consent was not obtained (e.g. the data were analyzed anonymously)   Animal Research (involving vertebrate animals, embryos or tissues)   - Provide the name of the Institutional Animal Care and Use Committee (IACUC) or other relevant ethics board that reviewed the study protocol, and indicate whether they approved this research or granted a formal waiver of ethical approval - Include an approval number if one was obtained - If the study involved non-human primates, add additional details about animal welfare and steps taken to ameliorate suffering - If anesthesia, euthanasia, or any kind of animal sacrifice is part of the study, include briefly which substances and/or methods were applied   Field Research  Include the following details if this study involves the collection of plant, animal, or other materials from a natural setting:   - Field permit number - Name of the institution or relevant body that granted permission |  |
| --- | --- |
|  |  |
| Data Availability  Authors are required to make all data underlying the findings described fully available, without restriction, and from the time of publication. PLOS allows rare exceptions to address legal and ethical concerns. See the [PLOS Data Policy](http://journals.plos.org/plosone/s/data-availability) and [FAQ](http://journals.plos.org/plosone/s/data-availability#loc-faqs-for-data-policy) for detailed information. | Yes - all data are fully available without restriction |

| A Data Availability Statement describing where the data can be found is required at submission. Your answers to this question constitute the Data Availability Statement and will be published in the article, if accepted. |  |
| --- | --- |
| Important: Stating ‘data available on request from the author’ is not sufficient. If your data are only available upon request, select ‘No’ for the first question and explain your exceptional situation in the text box. |  |
| Do the authors confirm that all data underlying the findings described in their manuscript are fully available without restriction? |  |
| Describe where the data may be found in full sentences. If you are copying our sample text, replace any instances of XXX with the appropriate details.   - If the data are held or will be held in a public repository, include URLs, accession numbers or DOIs. If this information will only be available after acceptance, indicate this by ticking the box below. For example: All XXX files   are available from the XXX database (accession number(s) XXX, XXX.).   - If the data are all contained within the manuscript and/or Supporting Information files, enter the following: All relevant data are within the manuscript and its Supporting Information files. - If neither of these applies but you are able to provide details of access elsewhere, with or without limitations, please do so. For example:   Data cannot be shared publicly because of [XXX]. Data are available from the XXX Institutional Data Access / Ethics Committee (contact via XXX) for researchers who meet the criteria for access to confidential data.  The data underlying the results presented in the study are available from (include the name of the third party | All relevant data are within the manuscript and its Supporting Information files |

| and contact information or URL).  • This text is appropriate if the data are owned by a third party and authors do not have permission to share the data.  * typeset |  |
| --- | --- |
| Additional data availability information: |  |

Manuscript	[Click here to access/download;Manuscript;Manuscript.docx](https://www.editorialmanager.com/pone/download.aspx?id=33576982&guid=96ba2594-8ce1-4a60-b5c6-dcfb18246b01&scheme=1)

1	**“The impact of the COVID-19 pandemic on research activities**

# 2 in the largest Italian academic community”

## 3 HOW PANDEMIC HAS CHANGED THE WORLD OF ACADEMIC RESEARCH 4 Massimo Volpe MD^1^, Massimo Ralli MD^2^, Andrea Isidori MD^3^

5

6 ^1^ Department of Clinical and Molecular Medicine, Faculty of Medicine and Psychology, Sapienza 7 University of Rome, Italy. ORCID 0000-0002-9642-8380

8	^2^ Department of Sense Organs, Faculty of Medicine, Sapienza University of Rome, Italy. ORCID 9	0000-0001-8776-0421

10	^3^ Department of Experimental Medicine, Faculty of Medicine, Sapienza University of Rome, Italy. 11	ORCID 0000-0002-9037-5417

12

13

14	Corresponding author: Massimo Volpe, Department of Clinical and Molecular Medicine, Sapienza 15	University of Rome, via di Grottarossa 1035-1039, 00189 Rome, Italy. +39(6)33775979; 16	massimo.volpe@uniroma1.it

17

18

19	Total word count: 6360 including tables

## Abstract (341 words)

Purpose: The aim of the present work is to explore the impact of the COVID-19 pandemic on research activities in a vast multidisciplinary academic community in order to identify the most critical issues.

Method: To this purpose we planned a survey addressed to the entire academic research staff at "*Sapienza*" University of Rome, which represents the largest Italian academic community. A questionnaire consisting of both open and closed questions was delivered to 4118 individuals in April 2021.

Results: 544 responses were collected. All academic roles were sufficiently represented in the research cohort. The median number of critical issues experienced by academic research staff was three. Among these, the three most frequently reported were related to: "Access to libraries / laboratories / research sites" (21.9%), "Limitation to stay abroad / study / research periods" (17.6%), "Progress of experimental work" (14.7%), with variable prevalence according to academic position and gender. Older subjects reported issues with "Projects’ financial reporting” and “Expiration of acquired consumable material more frequently”. The most common critical aspects reported in relation to the economic burden were: being “Unable to allocate funds” (31.4%), a “Reduction in clinical and scientific activity” (26.3%) and experiencing “Increased expenses (comprising private costs)” (21.2%) with no differences among genders. Researchers in Applied Sciences and Natural Sciences reported a higher frequency of problems in clinical and scientific activities, whereas increased expenses were reported also by researchers operating in the Humanities field. As a possible solution aimed at improving these critical issues, most subjects, especially those aged >45 years, indicated “Economic aid” (22.6%), “Reduction in bureaucracy” (19.9%) or “Enhancement of the scientific and clinical activities”, whereas those ≤45 years felt that an increased duration of and better access to PhD programmes were to be prioritized.

Conclusion: Our findings highlight the most critical issues related to research activities during

COVID-19 pandemic in a large academic community. The information achieved may be useful to

46	identify researchers’ needs and to design appropriate policies aimed at preparing research 47	institutions for unexpected catastrophic events and limiting the negative impact on academic

48	research activities.

# Introduction

Since early 2020, the outbreak of COVID-19 pandemic has disrupted not only societal and individual lifestyles around the world but has also heavily affected and impaired scientific research activities. Beside causing an enormous impact on healthcare systems, the pandemic outbreak caused by SARS-CoV-2 has, also heavily limited research programs in all academic fields by interfering with key steps of research activities at multiple levels [1]. A recent publication by Myers et al. [2] reported that since the onset of the COVID-19 pandemic and the consequent disruption of research activities, faculties have lost, on average, 24% of their research productivity. Laboratory-based scientists have been the most affected, as they lost up to 40% of their productivity. Indeed, many research institutions and universities from different countries severely reduced on-site academic activities [3]. A recent survey collecting data from more than 2000 subjects (scientists working in research and/or educational institutions, government agencies, industry, or other institutions involving science, technology, engineering, and mathematics [STEM]-related fields) across 132 countries reported delays in research activities and administrative duties, as well as increased workloads from adapting to online teaching environments [3]. The COVID-19 pandemic affected scientists in all fields [4], particularly in scientific fields that require laboratory resources, living animals, and time-sensitive experiments [5]. In this setting, healthcare research experienced the greatest impact. ~~Indeed,~~ in the UK, preliminary estimates suggest that over 1,500 academic trainees have been re-assigned exclusively to clinical activities during the pandemic [6]. Among the multiple consequences of the COVID-19 pandemic, closure of laboratories and research institutes, staff shortages, supply chain disruptions for reagents and laboratory materials as well as funding deficits have represented significant, often insurmountable, hardships for the "physiological" flow of research [7-10]. Moreover, COVID-19 pandemic has adversely affected clinical trials, which were often delayed or deferred [3]. An impressive shift of research activities towards those focused on

COVID-19 was also recorded [11]. In terms of publishing trends, some have reported a decrease in research quality and accuracy, premature publication, and a reduction in the space given to important non-COVID work [12].

In this general setting, the temporary shutdown of social relations during the pandemic, the increased caregiving responsibility for family, and the reduction of time available to work had an especially significant effect on women, who are often more involved in domestic responsibilities and childbearing duties [13-14].

Studies suggest that the pandemic exacerbated existing gender disparities for publications and career progress [15]. Indeed, numerous studies focused on disproportionate changes in publications between men and women during the pandemic [16].

Beside a general halt to research activities, emphasis has been placed on the effects on Ph.D. students, postdoctoral fellows and junior faculty, for whom the toll of lost research-time may be greater, as they are expected to produce their academic work and achieve research results within the limited timespan of their fellowships [[17].](https://paperpile.com/c/lwGxp5/NUxl) Consequently, early-career researchers (ECR) have experienced repercussions on careers and job opportunities, especially Ph.D. students and younger researchers [17-19].

At the opposite end of the spectrum in terms of consequences of the pandemic, some potentially positive "side effects", such as an acceleration of digitalization processes, adoption of remote work and remote connections, and a higher productivity in specific fields, such as artificial intelligence [20] ought to be mentioned.

The dramatic time experienced by the scientific community during COVID-19 pandemic has revealed the most fragile aspects of academic organizations and research activities, and the lessons learned should promote actions to respond and be prepared to preserve scientific activities during challenging times and emergencies.

Therefore, to explore the impact of the pandemic on research activities and in the attempt to identify the most critical issues, we performed a survey within our academic community based in

"*Sapienza*" University of Rome, ~~the largest European university by student number with ~117.000 students. At the time of the survey, our community included 3300 academics, 845 fixed-term researchers, and 2874 Ph.D. students, all involved in academic research activities. For its size and characteristics, Sapienza University is highly representative of a large multidisciplinary academic population, spanning through different ages, genders, and career stages and acting as an international environment that boosts international relationships and cooperation (with about 9% of international students).~~ To our knowledge, our current work is the first paper in Italy that attempts to identify the impact of COVID-19 pandemic on research activities of an entire academic community involving all areas and disciplines.

# Methods

For the scope of our research, the Governance Research Team of the University developed a questionnaire, consisting of both open-ended and closed-ended questions. It aimed to explore the differences in impact of the pandemic on different academic subgroups, on whom, based on the available literature, a higher toll from the pandemic might be expected. The closed-ended questions were related to the research field, the main factors influencing research activity during the COVID-19 pandemic, and the consequent financial impact of research activity limitations. The open-ended questions addressed the specific effects of the financial issues on research activity and required suggestions and feedback about emerging post-pandemic research priorities. The full questionnaire is attached as Supplementary Material 1. This questionnaire, proposed in Italian to participants, comprised the following items: age, sex, academic position, department, faculty, research type, main critical issues experienced during the pandemic, additional issues, absence/presence of an economic impact (and its entity), reasons underlying the economic burden (if any), and suggestions to improve academic research and performance. Sociodemographic and economic data was also collected: age, income, gender. The questionnaire was developed using Google Forms (Google LLC, USA). The protocol for the current survey has been approved by the Sapienza

Ethics Committee for Transdisciplinary Research (CERT) (approval no. CERT protocol ID 64/2023). Each individual participating in this survey has given explicit informed consent to the use of data. All data were collected in anonymous and aggregated form. The questionnaire was sent via email in April 2021 to all faculty members (full, associate, and assistant professors), Ph.D. students and medical residents of Sapienza University, which counts 4118 individual email addresses. Five hundred and forty-four individuals replied to the questionnaire, amounting to approximately 13,2% of the total invited cohort.

The academic position was classified into the following categories: "Ph.D. students", "Fixed-term researchers", "Full-term researchers", "Associate professors" and "Full professors".

We categorised all respondents according to their Department and Faculty into the following

Academic fields: Healthcare Professions and Applied Sciences, Humanities, Natural Sciences, Social Sciences and Formal Sciences. Regarding research type, the respondents could choose one among the following: Theoretical, Basic, Technical/Applied, Field, Clinical or Biological. Respondents were asked to specify the main and any other relevant issues affecting academic research during the pandemic, choosing within a list of 14 pre-specified possible answers. With regard to subjects experiencing an economic burden, the respondents could classify it according to the following classes: <10.000 euro, 10.000-30.000 euro, 30.000 – 60.000 euro, 60.000 – 100.000 euro and > 100.000 euro. Subjects were also asked to define the main reason underlying the experienced economic burden and to provide suggestions to restart academic research; these were open-ended questions, categorised into broad groups**.**

Responses were exported in an Excel database (Microsoft Corp, USA) and analysed using statistical software SPSS Statistics for Windows (version 27, IBM Corp).

Data distribution was visually inspected by analysing the respective histograms and normality plots. Data are presented as counts, percentages (%), means and standard deviations (SD), and medians with 25-75% interquartile ranges (IQR), as appropriate. Analyses were conducted employing_2_ tests for categorical variables, ANOVA, linear regression and logistic regression analyses were used for X variables.

Given the exploratory nature of the study focused on an objective, rather than on pre-specified hypotheses, we chose not to conduct post-hoc tests or to adjust for multiple comparisons [21-23]. A robust approach using bootstrapping for 2000 samples was employed to account for non-normal data distribution, and bias-corrected accelerated (BCa) 95% confidence intervals were calculated and reported. The linear regression analyses used Stein's formula for adjusted R squared (Adj R^2^) to evaluate how well the models cross-validate across different samples of data from the same population [12], while for logistic regression analyses, we reported R^2^ values according to Nagelkerke (R^2^_N_).

The level of statistical significance was set at a P-value <0.05. All statistical computations were conducted with the IBM SPSS Statistics for Windows (version 27, IBM Corp.). The full anonymized dataset underlying the findings described in this manuscript has been uploaded in Figshare (Figshare LLC, USA). DOI: 10.6084/m9.figshare.23551743

# Results

Among the recipients of the online questionnaire-based interview, 544 responses were collected. The descriptive characteristics of the research cohort are presented in Table 1 according to the academic position, and divided on the basis of the academic field, research type and economic burden experienced. Overall, 52,2% of the respondents were female and the median age was 48 years (35-57); all academic roles were adequately represented in the research cohort, thus reflecting a substantially well-balanced sample of Ph.D. students (22.2%), Fixed-term researchers (13.1%), Full-term researchers (13.1%), Associate professors (36.0%) and Full professors (15.6%).

176

177	**Table 1.** Description of the research cohort.

|  | **Total** | **PhD student** | **Fixed-term researcher** | **Full-term researcher** | **Associate professor** | **Full professor** |
| --- | --- | --- | --- | --- | --- | --- |
| n (%) | 544 | 121 (22,2) | 71 (13.1) | 71 (13.1) | 196 (36.0) | 85 (15.6) |
| Females (%) | 52.2 | 62.8 | 62 | 54.9 | 46.4 | 40 |
| Age (yrs, [25-75% IQR]) | 48 [35-57] | 28 [27-31] | 37.5 [34-44] | 49 [43-56] | 53 [47-59] | 59 [55-63] |
| **Academic field (%)** |  |  |  |  |  |  |
| Professions and app. sciences | 33.1 | 32.2 | 36.6 | 42.3 | 30.6 | 29.4 |
| Humanities | 22.8 | 24 | 15.5 | 12.7 | 22.4 | 17.6 |
| Natural sciences | 18.4 | 18.2 | 19.7 | 16.9 | 20.4 | 14.1 |
| Social sciences | 17.6 | 17.4 | 22.5 | 25.4 | 20.4 | 20 |
| Formal sciences | 8.1 | 8.3 | 5.6 | 2.8 | 6.1 | 18.8 |
| **Research type (%)** |  |  |  |  |  |  |
| Theoretical | 30.1 | 39.7 | 26.8 | 23.9 | 26 | 34.1 |
| Basic | 20.6 | 11.6 | 23.9 | 19.7 | 28.1 | 14.1 |
| Technical/Applied | 17.5 | 19 | 21.1 | 11.3 | 15.8 | 21.2 |
| Field | 13.2 | 10.7 | 9.9 | 16.9 | 15.3 | 11.8 |
| Clinical | 11.2 | 13.2 | 8.5 | 18.3 | 8.7 | 10.6 |
| Biological | 7.4 | 5.8 | 9.9 | 9.9 | 6.1 | 8.2 |
| **Economic burden (%)** |  |  |  |  |  |  |
| None | 68 | 67.8 | 76.1 | 69 | 68.9 | 58.8 |
| <10 000 euro | 11 | 14 | 8.5 | 14.1 | 10.7 | 7.1 |
| 10 000 - 30 000 euro | 7.9 | 1.7 | 2.8 | 5.6 | 10.2 | 17.6 |
| 30 000 - 60 000 euro | 1.8 | / | 2.8 | 1.4 | 1.5 | 4.7 |
| 60 000 - 100 000 euro | 0.6 | / | 1.4 | 1.4 | 0.5 | / |
| >100 000 euro | 0.4 | / | / | / | / | 2.4 |

178

179

180

181

182

Note: academic fields were categorized into the 5 major scientific branches, **Formal sciences** (mathematics, logic, statistics, computer science, etc.), **Natural sciences** (physics, chemistry, biology, earth sciences, etc.) and **Social sciences** (archaeology, geography, anthropology, psychology, sociology, economics, political science, cultural/ethnic studies, gender studies, etc.), and **Humanities** (literature, philosophy, theology, linguistics, human history, arts, etc.) and **Professions and applied sciences** (medicine, law, agriculture, education, physical performance, consumer science, journalism, military sciences, engineering, architecture and design, transportation, business, etc.).

## Research-related issues experienced

The respondents were asked to state the main issue affecting academic research experienced during the pandemic and to list all issues encountered. The description of the main and the general research-related issues is presented in Table 2.

**Table 2.** Description of the main and overall research-related issues experienced.

| Median number of issues experienced (IQR 25-75%) | 3 [2-4] |  |
| --- | --- | --- |
| List of issues experienced (n, %) | Main | Overall |
| Access to libraries / laboratories / research sites | 119 (21.9) | 264 (48.5) |
| Limitation to abroad stay /study / research periods | 96 (17.6) | 279 (51.3) |
| Advancement of experimental work | 80 (14.7) | 183 (33.6) |
| Scientific production | 63 (11.6) | 207 (38.1) |
| Family organization | 43 (7.9) | 144 (26.5) |
| Patients’ enrollment / access | 35 (6.4) | 83 (15.3) |
| Sources retrieval | 35 (6.4) | 130 (23.9) |
| Start of experimental work / obtainment of authorizations | 24 (4.4) | 87 (16) |
| Transport logistics | 14 (2.6) | 97 (17.8) |
| Limitation to supply of services (maintenance, etc.) | 14 (2.6) | 73 (13.4) |
| Contract expiry of personnel specifically recruited for research | 8 (1.5) | 50 (9.2) |
| Projects’ financial reporting | 6 (1.1) | 63 (11.6) |
| Access to calls with deadlines (awards, grants, etc.) | 5 (0.9) | 38 (7) |
| Expiry of acquired consumable material | 2 (0.4) | 34 (6.3) |

189

The median number of issues experienced was 3 (2-4). Among the main issues, the five most frequently encountered were related to: "Access to libraries / laboratories / research site" (21.9%), "Limitation to abroad stay / study / research periods" (17.6%), "Advancement of experimental work" (14.7%), "Scientific production" (11.6%) and "Family organisation" (7.9%). Interestingly, considering all research issues experienced, these five also represented the five most frequently encountered, albeit with a different frequency: "Limitation to abroad stay / study / research periods" (51.3%), "Access to libraries / laboratories / research sites" (48.5%), "Scientific production" (38.1%), "Advancement of experimental work" (33.6%) and "Family organisation" (26.5%).

Table 3 presents the frequency of research-related issues encountered according to their academic position. Specifically, "Access to libraries / laboratories / research sites" showed a significant difference in frequency, being highest among Ph.D. students (66.9%) and Fixed-term researchers

(50.7%), whereas a "Limitation to abroad stay / study / research periods" was reported especially by

Full professors (62.4%) and Ph.D. students (62%) and "Family organisation" was frequent among Associate professors (34.2%) and Full-term researchers (28.2%). On the other hand, no significant differences were observed regarding the frequency distribution of "Advancement of experimental work" and "Scientific production".

206 **Table 3.** Research issues experienced based on academic position

**PhD students**

**Fixed**

**-**

**term**

**researchers**

**Full**

**-**

**term**

**researchers**

**Associate**

**professors**

**Full**

**professors**

***p***

**n**

121

71

71

196

85

**List of overall issues experienced [n (%)]**

| Access to libraries / laboratories / research site | 81 (66.9) | 36 (50.7) | 28 (39.4) | 78 (39.8) | 41 (48.2) | **<0.0001** |
| --- | --- | --- | --- | --- | --- | --- |
| Limitation to abroad stay /study / research periods | 75 (62) | 33 (46.5) | 20 (28.2) | 98 (50) | 53 (62.4) | **<0.0001** |
| Advancement of experimental work | 42 (34,7) | 24 (33.8) | 23 (32.4) | 65 (33.2) | 29 (34.1) | 0.99 |
| Scientific production | 44 (36.4) | 31 (43.7) | 30 (42.3) | 80 (40.8) | 22 (25.9) | 0.11 |
| Family organization | 29 (24) | 17 (23.9) | 20 (28.2) | 68 (34.2) | 11 (12.9) | **0.005** |
| Patients’ enrolment / access | 16 (13.2) | 13 (18.3) | 15 (21.1) | 27 (13.8) | 12 (14.1) | 0.53 |
| Sources retrieval | 43 (35.5) | 20 (28.2) | 13 (18.3) | 41 (20.9) | 13 (15.3) | **0.004** |
| Start of experimental work / obtainment of authorizations | 31 (25.6) | 13 (18.3) | 9 (12.7) | 26 (13.3) | 8 (9.4) | **0.011** |
| Transport logistics | 24 (19.8) | 15 (21.1) | 14 (19.7) | 33 (16.8) | 11 (12.9) | 0.64 |
| Limitation to supply of services (maintenance, etc.) | 12 (9.9) | 8 (11.3) | 10 (14.1) | 26 (13.3) | 17 (20) | 0.31 |
| Contract expiry of personnel specifically recruited for research | / | 9 (12.7) | 6 (8.5) | 19 (9.7) | 16 (18.8) | **<0.001** |
| Projects’ financial reporting | 4 (3.3) | 12 (16.9) | 6 (8.5) | 28 (14.3) | 13 (15.3) | **0.01** |
| Access to calls with deadlines (awards, grants, etc.) | 7 (5.8) | 8 (11.3) | 5 (7) | 12 (6.1) | 6 (7.1) | 0.65 |
| Expiry of acquired consumable material | 3 (2.5) | 3 (4.2) | 5 (7) | 17 (8.7) | 6 (7.1) | 0.24 |

207 Note: significant *P*-values are presented in bold.

26

208 We further analysed the impact of gender, age and academic field on the reported research issues.
209 The full data are reported in Table 4.

210

**Table 4.** Research issues experienced based on sex, age (≤45/>45 years) and scientific field

|  | **Males** | **Females** | ***p*** | **≤45 yrs** | **>45yrs** | ***p*** | **PAS** | **Hum** | **NS** | **Soc. Sci.** | **For. Sci.** | ***p*** |
| --- | --- | --- | --- | --- | --- | --- | --- | --- | --- | --- | --- | --- |
| **n** | 260 | 284 |  | 241 | 303 |  | 180 | 108 | 100 | 112 | 44 |  |
| **List of overall issues experienced (%)** |  |  |  |  |  |  |  |  |  |  |  |  |
| Access to libraries / laboratories / research site | 48.1 | 48.9 | 0.84 | 55.2 | 43.2 | **0.01** | 38.3 | 88.9 | 35 | 49.1 | 20.5 | **<0.001** |
| Limitation to abroad stay /study / research periods | 49.2 | 53.2 | 0.36 | 53.1 | 49.8 | 0.45 | 41.1 | 69.4 | 50 | 49.1 | 56.8 | **<0.001** |
| Advancement of experimental work | 33.5 | 33.8 | 0.91 | 32.4 | 34.7 | 0.58 | 43.3 | 13 | 55 | 20.5 | 29.5 | **<0.001** |
| Start of experimental work / obtainment of authorizations | 16.9 | 15.1 | 0.55 | 20.3 | 12.5 | **0.01** | 22.8 | 5.6 | 20 | 16.1 | 4.5 | **<0.001** |
| Scientific production | 39.2 | 37 | 0.60 | 41.9 | 35 | 0.10 | 37.8 | 34.3 | 40 | 33 | 56.8 | 0.07 |
| Family organisation | 25.8 | 27.1 | 0.76 | 31.5 | 22.4 | **0.02** | 20 | 26.9 | 23 | 33 | 43.2 | **0.01** |
| Patients’ enrollment / access | 10.8 | 19.4 | **0.01** | 14.5 | 15.8 | 0.67 | 29.4 | 1.9 | 6 | 19.6 | / | **<0.001** |
| Sources retrieval | 23.8 | 23.9 | 0.98 | 27.4 | 21.1 | 0.09 | 14.4 | 49.1 | 5 | 38.4 | 6.8 | **<0.001** |
| Transport logistics | 20.4 | 15.5 | 0.13 | 20.7 | 15.5 | 0.11 | 15 | 19.4 | 29 | 11.6 | 15.9 | **0.012** |
| Limitation to supply of services (maintenance, etc.) | 11.9 | 14.8 | 0.35 | 10.8 | 15.5 | 0.11 | 12.2 | 5.6 | 30 | 8.9 | 11.4 | **<0.001** |
| Contract expiry of personnel specifically recruited for research | 7.7 | 10.6 | 0.26 | 7.1 | 10.9 | 0.12 | 8.9 | 5.6 | 14 | 10.7 | 4.5 | 0.20 |
| Projects’ financial reporting | 11.5 | 11.6 | 0.99 | 8.3 | 14.2 | **0.03** | 10 | 10.2 | 11 | 17 | 9.1 | 0.39 |
| Access to calls with deadlines (awards, grants, etc.) | 5.8 | 8.1 | 0.30 | 7.9 | 6.3 | 0.46 | 6.1 | 6.5 | 5 | 9.8 | 9.1 | 0.64 |
| Expiry of acquired consumable material | 5.8 | 6.7 | 0.68 | 3.7 | 8.3 | **0.03** | 9.4 | 1.9 | 7 | 4.5 | 6.8 | 0.12 |

Note: significant *P*-values are presented in bold. Academic fields were categorised into the 5 major scientific branches, **Formal sciences** (mathematics, logic, statistics, computer science, etc.), **Natural sciences** (physics, chemistry, biology, earth sciences, etc.) and **Social sciences** (archaeology, geography, anthropology, psychology, sociology, economics, political science, cultural/ethnic studies, gender studies, etc.), and **Humanities** (literature, philosophy, theology, linguistics, human history, arts, etc.) and **Professions and applied sciences** (medicine, law, agriculture, education, physical performance, consumer science, journalism, military sciences, engineering, architecture and design, transportation, business, etc.).

Abbreviations: ***PAS* =** Professions and Applied Sciences; ***Hum*** *=* Humanities; ***NS*** = Natural Sciences; ***Soc. Sci. =*** Social Sciences; ***For. Sci.*** = Formal sciences.

26

217 No significant differences were found regarding males vs. females in the reported issues, except for 218 "Patients’' enrolment / access" which was more frequent among females.

219 We divided the research cohort according to age into two groups, ≤45 (n = 241) vs >45 years of age
220 (n = 303). The younger cohort experienced significantly more difficulties with regard to "Access to
221 libraries / laboratories / research sites", "Start of experimental work / obtainment of authorizations" 222 and "Family organisation". In contrast, the older cohort had higher rates of issues with "Projects’
223 financial reporting” and “Expiry of acquired consumable material”.
224 Most research issues significantly differed in frequency among academic fields, complete results 225 are shown in Table 4.
226 A multiple regression analysis of the number of research-related issues experienced, considering
227 respondents’ age, gender, and academic position, found a younger age as the only significant 228 independent predictor of a larger sum of overall issues reported (Table 5).

229

230

231 **Table 5.** Linear model of predictors of the number of research-related issues experienced.

***b***

**SE**

***β***

***p***

**Research-related issues number**

| Constant |  | 3.82 (3.31, 4.42) |  | 0.30 |  |  |  | <0.001 |
| --- | --- | --- | --- | --- | --- | --- | --- | --- |
| Age, years |  | -0.022 (-0.04, -0.01) |  | 0.01 |  | -0.21 |  | **0.008** |
| Sex, male |  | 0.11 (-0.12, 0.34) |  | 0.12 |  | 0.04 |  | 0.35 |
| Academic position |  | -0.05 (-0.04, -0.15) |  | 0.05 |  | 0.09 |  | 0.25 |

232 *Note:* 95% bias corrected accelerated (BCa) confidence intervals, SE and *P* values from 2000 bootstrapped samples are reported in 233 parentheses. Significant *P*-values are presented in bold. *P* for the model = 0.006; Adj R^2^ = 0.01.

234	Abbreviations: ***β*** = standardized coefficient; ***b*** = unstandardized coefficient; **SE =** standard error.

## Experience and causes of Economic burden

Overall, 117 respondents (21.7%) reported the presence of an economic burden deriving from the pandemic, with no significant differences according to gender, age group or academic field. In half of the cases reporting an economic burden, it was below 10 000 euros. Complete results are presented in Table 6.

26

240 **Table 6.** Presence of economic burden, causes and suggestions to improve research according to sex, age range (45/>45 years) and scientific field.

|  | **Total** | **Males** | **Females** | ***p*** | **≤45yrs** | **>45yrs** | ***p*** | **PAS** | **Hum** | **NS** | **Soc. Sci.** | **For. Sci.** | ***p*** |
| --- | --- | --- | --- | --- | --- | --- | --- | --- | --- | --- | --- | --- | --- |
| **n** | 544 | 260 | 284 |  | 241 | 303 |  |  |  |  | 96 | 44 |  |
| **Presence of any economic burden (%)** | 21.7 | 25 | 18.7 | 0.07 | 19.5 | 23.4 | 0.27 |  |  |  | 13.5 | 13.6 | 0.13 |
| **List of reasons for economic burden (%)** |  |  |  |  |  |  |  |  |  |  |  |  |  |
| Unable to allocate funds | 31.4 | 27.7 | 35.8 | 0.19 | 28.6 | 38.8 | 0.28 | 30.2 | 28 | 47.8 | 33.3 | 50 | 0.52 |
| Reduction in clinical and scientific activity | 26.3 | 30.8 | 20.8 | 0.19 | 19 | 35.8 | 0.06 | 39.5 | 8 | 39.1 | 16.7 | 33.3 | **0.046** |
| Increased expenses (comprising private costs) | 21.2 | 21.5 | 20.8 | 0.99 | 31 | 17.9 | 0.12 | 27.9 | 40 | / | 25 | / | **0.01** |
| Interrupted mobility programmes | 5.9 | 6.2 | 5.7 | 0.95 | 9.5 | 4.5 | 0.30 | 2.3 | 8 | 4.3 | 25 | / | 0.07 |
| Reduction in the number of calls and grants available | 2.5 | 3.1 | 1.9 | 0.71 | 2.4 | 3 | 0.85 | / | 8 | / | / | 16.7 | 0.06 |
| Closure of archives and libraries | 1.7 | 1.5 | 1.9 | 0.86 | 4.8 | / | 0.07 | / | 8 | / | / | / | 0.14 |
| Lack of personnel | 1.7 | 1.5 | 1.9 | 0.86 | 4.8 | / | 0.07 | / | / | 8.7 | / | / | 0.11 |
| **Suggestions to improve academic research (%)** |  |  |  |  |  |  |  |  |  |  |  |  |  |
| Economic aid | 22.6 | 19.4 | 25.2 | 0.24 | 18.6 | 25.1 | 0.19 | 23 | 13.2 | 38.5 | 24 | 10.5 | **0.01** |
| Reduction in bureaucracy | 19.9 | 23.9 | 16.6 | 0.12 | 12.7 | 24.6 | **0.012** | 19 | 15.8 | 21.2 | 18 | 42.1 | 0.14 |
| Enhancement of the scientific and clinical activities | 19.2 | 20.1 | 18.4 | 0.71 | 18.6 | 19.6 | 0.85 | 25 | 11.8 | 13.5 | 24 | 21.1 | 0.15 |
| Increasing the PhD length and ease of access | 11.1 | 8.2 | 13.5 | 0.15 | 22.9 | 3.4 | **<0.00**    **1** | 14 | 6.6 | 11.5 | 14 | 5.3 | 0.48 |
| Digitalization | 9.8 | 11.9 | 8 | 0.25 | 8.5 | 10.6 | 0.54 | 7 | 22.4 | 3.8 | 6 | / | **0.001** |
| Resumption of presence activities | 6.4 | 6.7 | 6.1 | 0.84 | 3.4 | 8.4 | 0.09 | 4 | 6.6 | 7.7 | 6 | 15.8 | 0.42 |
| Re-opening of archives and libraries | 5.7 | 5.2 | 6.1 | 0.74 | 10.2 | 2.8 | **0.008** | 2 | 15.8 | / | 6 | / | **<0.00**  **1** |
| Increasing the personnel | 3 | 2.2 | 3.7 | 0.47 | 2.5 | 3.4 | 0.69 | 5 | 3.9 | 1.9 | / | / | 0.42 |
| Enhancement of abroad mobility programmes | 2.4 | 2.2 | 2.5 | 0.90 | 2.5 | 2.2 | 0.86 | 1 | 3.9 | 1.9 | 2 | 5.3 | 0.66 |

241 Note: significant *P*-values are presented in bold. Academic fields were categorised into the 5 major scientific branches, **Formal sciences** (mathematics, logic, statistics, computer science, etc.), **Natural sciences** 242 (physics, chemistry, biology, earth sciences, etc.) and **Social sciences** (archaeology, geography, anthropology, psychology, sociology, economics, political science, cultural/ethnic studies, gender studies, etc.), and 243 **Humanities** (literature, philosophy, theology, linguistics, human history, arts, etc.) and **Professions and applied sciences** (medicine, law, agriculture, education, physical performance, consumer science, 244 journalism, military sciences, engineering, architecture and design, transportation, business, etc.).
245 Abbreviations: ***PAS* =** Professions and Applied Sciences; ***Hum*** *=* Humanities; ***NS*** = Natural Sciences; ***Soc. Sci. =*** Social Sciences; ***For. Sci.*** = Formal sciences.

26

246 The three most frequently reported reasons underlying an economic burden were: being “Unable to
247 allocate funds” (31.4%), a “Reduction in clinical and scientific activity” (26.3%) and experiencing
248 “Increased expenses (comprising private costs)” (21.2%). No specific differences were reported
249 between males and females or between younger and older respondents. However, those in the fields 250 of Healthcare Professions and Applied Sciences and Natural Sciences reported a higher frequency 251 of problems in clinical and scientific activities (39.5% and 39.1%, respectively)~~,~~ while increased 252 (also personal) expenses were reported by those in the Humanities field (40%).
253 When analysed according to academic role, Ph.D. students reported “Increased expenses
254 (comprising private costs)” more frequently when compared to other groups (38.9%), and they were
255 the only category to mention “Closure of archives and libraries” as problematic (11.1%). Full 256 results are reported in Table 7.

26

257 **Table 7.** Presence of economic burden, causes and suggestions to improve research according to academic position.

|  | **PhD student** | **Fixed-term researcher** | **Full-term researcher** | **Associate professor** | **Full professor** | ***p*** |
| --- | --- | --- | --- | --- | --- | --- |
| **n** | 121 | 71 | 71 | 196 | 85 |  |
| **Presence of any economic burden (%)** | 15.7 | 15,5 | 22,5 | 23 | 31.8 | 0.05 |
| **List of reasons for economic burden (%)** |  |  |  |  |  |  |
| Unable to allocate funds | 22.2 | 60 | 26.7 | 33.3 | 40.7 | 0.30 |
| Reduction in clinical and scientific activity | 16.7 | 20 | 20 | 30.8 | 44.4 | 0.25 |
| Increased expenses (comprising private costs) | 38.9 | 10 | 40 | 23.1 | 7.4 | **0.044** |
| Interrupted mobility programmes | 11.1 | / | / | 10.3 | 3.7 | 0.47 |
| Reduction in the number of calls and grants available | / | 10 | 6.7 | / | 3.7 | 0.35 |
| Closure of archives and libraries | 11.1 | / | / | / | / | **0.034** |
| Lack of personnel | / | / | 6.7 | 2.6 | / | 0.56 |
| **Suggestions to improve academic research (%)** |  |  |  |  |  |  |
| Economic aid | 16.4 | 25 | 25.7 | 23.5 | 24.6 | 0.75 |
| Reduction in bureaucracy | 10.4 | 5.6 | 20 | 29.4 | 22.8 | **0.005** |
| Enhancement of the scientific and clinical activities | 17.9 | 19.4 | 14.3 | 18.6 | 24.6 | 0.80 |
| Increasing the PhD length and ease of access | 31.3 | 11.1 | 2.9 | 5.9 | 1.8 | **<0.001** |
| Digitalization | 4.5 | 13.9 | 17.1 | 9.8 | 8.8 | 0.29 |
| Resumption of presence activities | 4.5 | 5.6 | 8.6 | 5.9 | 8.8 | 0.86 |
| Re-opening of archives and libraries | 10.4 | 11.1 | 2.9 | 2.9 | 3.5 | 0.13 |
| Increasing the personnel | 1.5 | 8.3 | 5.7 | 2 | 1.8 | 0.24 |
| Enhancement of abroad mobility programmes | 3 | / | 2.9 | 2 | 3.5 | 0.84 |

258 Note: significant *P*-values are presented in bold. Academic fields were categorized into the 3 major scientific branches, **Formal sciences** (mathematics, logic, statistics, computer science, etc.), **Natural**
259 **sciences** (physics, chemistry, biology, earth sciences, etc.) and **Social sciences** (archaeology, geography, anthropology, psychology, sociology, economics, political science, cultural/ethnic studies, gender 260 studies, etc.), and **Humanities** (literature, philosophy, theology, linguistics, human history, arts, etc.) and **Professions and applied sciences** (medicine, law, agriculture, education, physical performance, 261 consumer science, journalism, military sciences, engineering, architecture and design, transportation, business, etc.).

262	Abbreviations: ***PAS* =** Professions and Applied Sciences; ***Hum*** *=* Humanities; ***NS*** = Natural Sciences; ***Soc. Sci. =*** Social Sciences; ***For. Sci.*** = Formal sciences.

26

In a logistic regression analysis on the presence of economic burden, comprising age, gender and academic role, only the position of Full professor was found to be a significant independent predictor of the presence of (any) economic burden **(**Table 8**)**.

**Table 8.** Logistic regression analysis of the presence of economic burden.

***b***

**SE**

***β***

***p***

**Presence**

**of economic burden**

Constant

-

0.93 (

-

1.99

,

0.03)

0.56

0.09

| Age, years |  | -0.02 (-0.05, 0.01) |  | 0.02 |  | 0.98 |  | 0.21 |
| --- | --- | --- | --- | --- | --- | --- | --- | --- |
| Sex, male |  | -0.30 (-0.74, 0.16) |  | 0.23 |  | 0.74 |  | 0.18 |
| Fixed-term researcher |  | 0.17 (-0.82, 1.04) |  | 0.47 |  | 1.18 |  | 0.70 |
| Full-term researcher |  | 0.78 (-0.26, 1.81) |  | 0.52 |  | 2.19 |  | 0.11 |
| Associate professor |  | 0.87 (-0.13, 1.91) |  | 0.47 |  | 2.38 |  | 0.07 |
| Full professor |  | 1.36 (0.11, 2.62) |  | 0.57 |  | 3.89 |  | **0.016** |

*Note:* 95% bias corrected accelerated (BCa) confidence intervals, SE and *P* values from 2000 bootstrapped samples are reported in parentheses. Significant *P*-values are presented in bold. *P* for the model = 0.06; R^2^_N_ = 0.034.

Abbreviations:	***β***	=	standardized	coefficient;	***b***	=	unstandardized	coefficient;	**SE	=**	standard	erro

26

## Suggestions to support academic research

Respondents were asked to provide suggestions on how to improve and “recover” academic research status; answers were categorised into nine broad groups and the results are reported in Table 6.

The three most frequent suggestions were the need of an “Economic aid” (22.6%), asking for a

“Reduction in bureaucracy” (19.9%), or the “Enhancement of the scientific and clinical activities” (19.2%).

No significant differences were reported according to gender. Those ≤45 years of age felt that increasing the length and improving the ease of access of the Ph.D. programme (22.9% vs 3.4%) was more necessary, together with re-opening archives and libraries (10.2% vs. 2.8%), while those >45 years of age suggested a reduction in bureaucracy more frequently (24.6% vs 12.7%).

Among the different academic fields, Natural Sciences requested more “Economic aid” while Humanities favoured a larger adoption of “Digitalization” and a prompt “Re-opening of archives

and libraries”.

A “Reduction in bureaucracy” was advocated especially by Associate and Full professors (29.4% and 22.8%, respectively), and unsurprisingly, PhD students suggested “Increasing the PhD length and ease of access” (31.3%). The full results are reported in Table 6.

# Discussion

The global storm that hit human activities during COVID-19 pandemic has heavily and broadly affected academic research activities. Although several previous reports have proposed analyses mostly focused on the hardships in specific fields, such as science and medicine, other important aspects have been substantially neglected. A comprehensive exploratory survey, encompassing research difficulties in different disciplines and at different career stages, was lacking in Europe.

For this reason, we investigated a representative sample of the academic population of “*Sapienza*” University with a web-based questionnaire. This study presents a unique, qualitative data-driven representation of the impact of COVID-19 on academic research activities in Italy. The results of our survey highlight several critical issues that influenced research activities and could contribute to develop future strategies to limit the ~~heavy~~ consequences of pandemics or other catastrophic events in the future, both in Italy and in other ~~European~~ countries with similar academic systems.

A first conclusion that can be drawn from the analysis of our survey is that most of the pre-existing drawbacks and barriers to academic activity have been amplified during the COVID-19 pandemic. In fact, the responses to the questionnaires reflected several weaknesses exacerbated by the pandemic which may require attention and preventive measures. Respondents reported a median of 3 research-related critical issues, with no significant differences among gender, age groups, academic position, research field or type. However, when accounting for age, gender and academic position, younger age was independently associated with a higher number of experienced critical issues (Table 5**)**. When asked about the main issue impacting research, difficulties concerning impaired access to research facilities (such as laboratories, libraries and research sites) emerged as the most frequent (21.9%) among respondents. This issue, although common to many (overall 48.5%) and in all academic fields, was significantly more prevalent among Ph.D. students (66.9%), younger researchers (55.2%) and extremely frequent in the field of Humanities (88.9%). This issue has been widely recognized as a main problem to research activities, especially scientific, during the pandemic. Often the typical worksite has been replaced by working at home in inadequate environmental conditions for the younger populations [24]. The impact on clinical science was also significantly affected due to many clinical trials having been paused or terminated due to pandemic restrictions, quarantines and lockdowns; similarly, patient enrolment for new studies has been frequently halted. These circumstances have prompted a specific set of publications from the US *Food and Drug Administration [U.S. Department of Health and Human Services, Food and Drug Administration. FDA guidance on conduct of clinical trials of medical products during COVID-19 public health emergency: guidance for industry, investigators, and institutional review boards. https://www.fda.gov/media/136238/download]* and from the European Medical Agency *[Guidance on the management of clinical trials during the COVID-19 (Coronavirus) pandemic*

[*https://ec.europa.eu/health/system/files/2021-02/guidanceclinicaltrials_covid19_en_0.pdf]*](https://ec.europa.eu/health/system/files/2021-02/guidanceclinicaltrials_covid19_en_0.pdf) guidelines on the conduct of clinical trials during the COVID-19 pandemic to preserve trial integrity, to ensure compliance and to assure subjects’ and patients’ safety.

On the other hand, when considering all problems encountered, travel limitations restricting abroad stay, study and/or research periods were felt as the most frequent issue overall (51.3%), despite having been raised as the main issue by only 17.6% of respondents. Ph.D. students, alongside full professors, reported this issue the most (62 and 62.4%, respectively), with the highest rates in the fields of Humanities (69.4%) and Formal Sciences (56.8%). This limitation has also affected scientific exchange, which has produced a global shift of many international conferences into virtual platforms for teaching and knowledge dissemination, thus somewhat limiting the opportunities for networking [17] and producing effects and “technostress” burnout [18]. A decrease in scientific contacts and networks during the pandemic, with subsequent impaired opportunities for interactions and peer support, has been reported [11]. On the other hand, global scientific collaboration has reached unprecedented levels, owing to data sharing about COVID-19 related analyses. Although fruitful, such endeavours have raised numerous issues including how to uphold the basic standards of scientific conduct and integrity [12]. Our survey also focused on the incidence of pandemic-associated economic burden on academics. Some degree of burden was reported approximately by 1 out of 5 respondents, with no significant differences according to gender, age or academic field. Inability to allocate research funds and a reduction in clinical activities were the most common reasons behind this burden. Indeed, with the prioritisation of COVID-19 studies, new clinical trials have been paused, with detrimental impacts on clinical academics involved in non-COVID 19 research [25]. However, increased expenses, also accounting for private costs, were also significantly present among Ph.D. students and researchers, especially in the field of Humanities. In a multiple logistic regression analysis to identify the predictors of an economic burden only the role of Full professor was independently associated with (any) economic burden, possibly because of the larger amount of funds managed. Part of our findings are in accordance with published literature, which reports a higher impact on Early Career Researcher (ECR) and on “wet laboratory researchers” [26-27]. As PhD students’ and fixed-term researchers’ positions are usually based on fellowship programmes lasting a limited amount of time, the lost research time has reasonably determined a heavy toll on these researchers, as they are expected to produce their scientific work and reach their research objectives within a limited time span [28]. Consequently, this academic population is particularly vulnerable to the impact of these conditions on their research output, because of delays in publication, partly deriving from difficulties in completing experimental work.

All these factors may reduce ~~smaller~~ opportunities for ECR’s funding applications and for job applications, in the current context of job cuts and hiring freeze due to global financial pressures. On the other hand, many journals have granted deadline extensions for submitting academic work, financial reporting of ongoing projects has been postponed by some research institutions and universities, and the time limit for the expense of funding derived from grants has often been extended. ~~Wet~~ researchers were particularly affected from the closure of laboratories and research institutions, as the loss of experimental work has been estimated to be between 1 and 6 months in approximately 25% of cases [2], compared to “dry research” that can be at least in part conducted remotely. Specifically, as previously reported, researchers working with cell cultures suffered widespread supply shortages concerning (e.g. qPCR) reagents and consumables (especially plasticware), but also including personal protective equipment (PPE), such as gloves and masks [3,7]. Similarly, researchers working with laboratory animals also experienced difficulties deriving from staff shortages, disruption in the supply chain of ~~veterinary~~ drugs, necessary tools for animal care and housing [2,7].

Our results partly differ from the available literature. No significant differences were found in the rates of research-related issues among male and female researchers, except for greater difficulties in women concerning patients’ enrolment and access, possibly due to a higher number of female physicians in our sample. Regarding researchers experiencing an economic burden, no significant differences were found between male and female, with a trend favouring females.

An equal rate of men and women experiencing difficulties with family organisation impacting their research activities emerged from our survey (25.8% vs 27.1%, respectively). This contrasts with published literature and reports focusing on the risks intrinsic to gender inequality in the academic context. In fact, female academics have been reported to more likely assume parenting or domestic responsibilities than their male counterparts. Furthermore, female researchers are more likely to shoulder domestic duties following the closure of universities and research institutions, as well as childbearing and home-schooling when lockdowns also involved schools and kindergartens [29].

Indeed, it has been postulated that many women are currently doing ‘second and third shifts’ regarding housework and welfare after their first shift of paid work [30]. The possible explanation of our data relies on the lack of information on family composition and the presence of offspring from participants in our survey, as parents are expected to suffer more significantly from the issues mentioned above [24]. Moreover, we do not have data about how the number of working hours has changed during the pandemic for male and female researchers. Indeed, data suggest a differential amount of available working hours for male/female and parents/not parents researchers [3]. Recent evidence suggest that gender-related issues emerged during COVID-19 have lasted well beyond the first wave. In 2020-2021 gender inequalities for women working in biology, biochemistry, and civil and environmental engineering at universities, have remained steady or worsened. Compared to men, a greater proportion of women who responded to surveys reported inability to focus on their research activities. Moreover, female scientists were also more likely to report having grant disruptions and research grants that face financial difficulties due to the pandemic. Furthermore, women make up only a third of authors named on COVID-19 related publications, and they are particularly underrepresented among first and last authorship positions [31]. An analysis on more than 2000 Elsevier journals during the first wave of the pandemic has shown that women submitted proportionally fewer manuscripts than men. This deficit was especially pronounced among junior cohorts of women academics [32]. This gender-related issue, requires more extensive analysis, since our current survey may not produce sufficient information.

As our work aimed at identifying key issues and possible ~~resilience~~ strategies that could help resume academic activities after the pandemic, we asked our community for suggestions. Notably, economic aid was felt necessary by 22.6%, especially by respondents in the field of Natural Sciences (38.5%). In contrast, older researchers favoured a reduction in bureaucracy in approximately a quarter of cases, Ph.D. students unsurprisingly suggested to increase the Ph.D. length and ease of access (31.3%) and Humanities researchers supported the need for a more widespread process of digitalization (22.4%) and advocated for the re-opening of archives and libraries (15.8%).

## Limitations

The present survey consisted of closed- and open-ended questions sent by email to people involved in research activities in Sapienza University. Some elements were not intended to be investigated in the survey, such as the quantification of the amount of time dedicated to research and different activities (teaching, mentoring etc) during the pandemic as well as the quality of remote work from home. Furthermore, our approach was focused on the subjective perceptions of the impact and consequences of COVID-19 on their own research, in qualitative rather than quantitative terms. Moreover, the composition of the family of respondents was not assessed, thus limiting the chance to derive conclusions about issues specifically related to parenthood. Lastly, it is unclear whether the findings and the conclusions drawn from our study may be similarly applied to other academic institutions in Italy or in Europe. However, our study has been conducted in the largest Italian University and one of the largest academic communities in Europe and its findings are largely consistent with previous similar studies.

## Conclusions

To our knowledge, this is the first thorough exploratory analysis of the impact of the COVID-19 pandemic on a population sample such as that included in our survey, which represents the whole active academic multidisciplinary research community in a large University. This provides many new ~~potential useful~~ insights for planning systematic changes to scientific institutions before and during new possible global crises. We had a heterogeneous study population in terms of position, rank, career stage, tenure status, discipline, and characteristics of work. Our results may represent a significant reference for policy makers, university administrators, and scientists about issues exacerbated by COVID-19 related policies and suggest a fundamental need to develop approaches future crises. Indeed, our findings, beside their potential intrinsic informative value, may be useful to design appropriate policies aimed at limiting the impact of unexpected catastrophic events on research, pointing out what could be needed to preserve and even reinforce research facilities and activities in critical situations like those caused by the SARS-COV-2 pandemic.

**Disclosures:**

Acknowledgements: The authors are grateful to the members of Sapienza Governance Research

Team: Mariasabrina Sarto, Francesca Bozzano, Carlo Catalano, Luciano Galantini, Marco Oliverio, Daniele Riccioni, Mauro Valorani, Antonella Cammisa, Ciro Franco, Andrea Riccio, Fabio Sciarrino.

Francesco Carlomagno, MD, PhD and Allegra Battistoni MD, PhD gave a substantial contribution in the development of the manuscript.

Melwyn Luis Muthukkattil, native English language speaker, has revised the manuscript.

Other disclosures: None

Ethical approval: Not applicable

Disclaimers: None

Previous presentations: None

Data sharing: no additional data

**S1 Appendix: Questionnaire in English**

### References

1. Sohrabi C , Mathew G, Franchi T, Kerwan A, Griffin M, Del Mundo JSC et al. Impact of the coronavirus (COVID-19) pandemic on scientific research and implications for clinical academic training – A review. Int J Surg*.* 2021; 86:57–63
2. Carr RM, Lane-Fall MB, South E, Brady D, Momplaisir F, Guerra CE, et al. Academic careers and the COVID-19 pandemic: Reversing the tide. Sci Transl Med. 2021;13:eabe7189.
3. Heo S, Chan AY, Diaz Peralta P, Jin L, Pereira Nunes CR, Bell ML. Impacts of the COVID-19 pandemic on scientists' productivity in science, technology, engineering, mathematics (STEM), and medicine fields. Humanit Soc Sci Commun. 2022; 9:434
4. Myers KR, Tham WY, Yin Y, Cohodes N, Thursby JG, Thursby MC, Schiffer P, Walsh JT,

Lakhani KR, Wang D, Unequal effects of the COVID-19 pandemic on scientists. Nat. Hum.

Behav. 2020; 4: 880–883

5. Mehta N, Inamdar V, Puthillam A et al. Assessing the impact of COVID-19 on STEM (science, technology, engineering, mathematics) researchers in India. Wellcome Open Res 2020; 7:157.
6. National Institute for Health Research, Progressing UK clinical academic training in 2020: addressing the challenges of COVID-19. https://www.nihr.ac.uk/document s/progressing-ukclinical-academic-training-in-2020-addressing-the-challenges-ofcovid-19/24958, 2020
7. Korbel JO, Stegle O. Effects of the COVID-19 pandemic on life scientists. Genome Biol*.* 2020;

21:1–5.

8. Grimm D. Respirators, quarantines, and worst-case scenarios: Lab animal facilities grapple with the pandemic. Science. 2020 Mar 18; Available from:

https://www.sciencemag.org/news/2020/03/respirators-quarantines-and-worst-case-scenarioslab-animal-facilities-grapple-pandemic

9. Madhusoodanan J. Frozen cells and empty cages: researchers struggle to revive stalled experiments after the lockdown. *Nature*. 2020 Jun 5. doi: 10.1038/d41586-020-01704-y.;

Available from: <https://www.nature.com/articles/d41586-020-01704-y>

10. Rimmer A. Covid-19: How clinical academic trainees came back to the wards en masse. *BMJ*.

2021; 373:n558. doi: 10.1136/bmj.n558

11. Sohrabi C, Mathew G, Franchi T, Kerwan A, Griffin M, Soleil C Del Mundo J, Ali SA, Agha M, Agha R. Impact of the coronavirus (COVID-19) pandemic on scientific research and implications for clinical academic training - A review. Int J Surg. 2021; 86:57-63.
12. Smith EMR, Rakestraw C, Farroni JS. Research integrity during the COVID-19 pandemic:

Perspectives of health science researchers at an Academic Health Science Center. Account Res.

2022:1-22. Epub ahead of print

13. Staniscuaski F, Reichert F, Werneck FP, de Oliveira L, Mello-Carpes PB, Soletti RC, et al.

Impact of COVID-19 on academic mothers. Science. 2020; 368:724.

14. Malisch JL, Harris BN, Sherrer SM, Lewis KA, Shepherd SL, McCarthy PC, et al. Opinion: In the wake of COVID-19, academia needs new solutions to ensure gender equity. Proc Natl Acad Sci U S A. 2020; 117:15378–15381.
15. Staniscuaski F, Kmetzsch L, Soletti RC, Reichert F, Zandonà E, Ludwig ZMC,et al Gender,

Race and Parenthood Impact Academic Productivity During the COVID-19 Pandemic: From

Survey to Action. Front Psychol. 2021; 12:663252

16. Bell ML, Fong KC. Gender Differences in First and Corresponding Authorship in Public Health Research Submissions During the COVID-19 Pandemic. Am J Public Health. 2021; 111:159163.
17. Paula JR. Lockdowns due to COVID-19 threaten PhD students’ and early-career researchers’ careers. Nature Ecology & Evolution. Nat Ecol Evol*.* 2020; 4:999
18. Woolston C. Pandemic darkens postdocs’ work and career hopes. Nature. 2020; 585:309-312.
19. Woolston C. Junior researchers hit by coronavirus-triggered hiring freezes. Nature*.*

2020;582:449–450.

20. Sreepadmanabh M, Sahu AK, Chande A. COVID-19: Advances in diagnostic tools, treatment strategies, and vaccine development. J Biosci. 2020; 45:148.
21. Groenwold RHH, Goeman JJ, Cessie SL, Dekkers OM. Multiple testing: when is many too much? Eur J Endocrinol. 2021; 184: E11-E14
22. Ranstam J. Multiple P-values and Bonferroni correction. Osteoarthritis Cartilage. 2016; 24:763-

764

23. Bender R, Lange S. Adjusting for multiple testing--when and how? J Clin Epidemiol.

2001;54:343-9

24. Heo S, Chan AY, Diaz Peralta P, Jin L, Pereira Nunes CR, Bell ML. Impacts of the COVID-19 pandemic on scientists' productivity in science, technology, engineering, mathematics (STEM), and medicine fields. Humanit Soc Sci Commun. 2022; 9:434
25. Finn GM, Crampton P, Buchanan JA, Balogun AO, Tiffin PA, Morgan JE, et al. The impact of the COVID-19 pandemic on the research activity and working experience of clinical academics, with a focus on gender and ethnicity: a qualitative study in the UK. BMJ Open.

2022; 12:e057655

26. Bishr Omary M, Hassan M. Opinion: Here’s how we restore productivity and vigor to the biomedical research workforce in the midst of COVID-19*.* Proc Natl Acad Sci U S A. 2020;

117:19612–19614.

27. Byrom N. COVID-19 and the Research Community: The challenges of lockdown for earlycareer researchers. eLife. 2020; 9: e59634. Published online 2020 Jun 12. doi:

10.7554/eLife.59634

28. Termini CM, Traver D. Impact of COVID-19 on early career scientists: an optimistic guide for the future. BMC Biol. 2020; 18:95.
29. Gewin V. Pandemic burnout is rampant in academia. Nature. 2021; 591:489–491
30. Chung H. Return of the 1950s housewives? how to stop coronavirus lockdown reinforcing sexist gender roles, 2020. Available: https:// theconversation.com/return-of-the-1950s housewife-how-to-stopcoronavirus-lockdown-reinforcing-sexist-gender-roles-134851.
31. Pinho-Gomes AC, Peters S, Thompson K, Hockham C, Ripullone K, Woodward M, et al. Where are the women? Gender inequalities in COVID-19 research authorship. BMJ Glob

Health. 2020; 5:e002922

32. Squazzoni F, Bravo G, Grimaldo F, García-Costa D, Farjam M, Mehmani B. Gender gap in journal submissions and peer review during the first wave of the COVID-19 pandemic. A study on 2329 Elsevier journals. PLoS One. 2021; 16:e0257919

Supporting Information

[Click here to access/download](https://www.editorialmanager.com/pone/download.aspx?id=33576984&guid=620d9a82-ce6d-41b5-979f-f5c8235a80a1&scheme=1)

[Supporting Information S1 appendix.docx](https://www.editorialmanager.com/pone/download.aspx?id=33576984&guid=620d9a82-ce6d-41b5-979f-f5c8235a80a1&scheme=1)

Revised Manuscript with Track Changes

1	**“The impact of the COVID-19 pandemic on research activities**

### 2 in the largest Italian academic community”

#### 3 HOW PANDEMIC HAS CHANGED THE WORLD OF ACADEMIC RESEARCH 4 Massimo Volpe MD^1^, Massimo Ralli MD^2^, Andrea Isidori MD^3^

5

6 ^1^ Department of Clinical and Molecular Medicine, Faculty of Medicine and Psychology, Sapienza 7 University of Rome, Italy. ORCID 0000-0002-9642-8380

8	^2^ Department of Sense Organs, Faculty of Medicine, Sapienza University of Rome, Italy. ORCID 9	0000-0001-8776-0421

10	^3^ Department of Experimental Medicine, Faculty of Medicine, Sapienza University of Rome, Italy. 11	ORCID 0000-0002-9037-5417

12

13

14	Corresponding author: Massimo Volpe, Department of Clinical and Molecular Medicine, Sapienza 15	University of Rome, via di Grottarossa 1035-1039, 00189 Rome, Italy. +39(6)33775979; 16	massimo.volpe@uniroma1.it

17

18

19	Total word count: 6360 including tables

#### Abstract (341 words)

Purpose: The aim of the present work is to explore the impact of the COVID-19 pandemic on research activities in a vast multidisciplinary academic community in order to identify the most critical issues.

Method: To this purpose we planned a survey addressed to the entire academic research staff at "*Sapienza*" University of Rome, which represents the largest Italian academic community. A questionnaire consisting of both open and closed questions was delivered to 4118 individuals in April 2021.

Results: 544 responses were collected. All academic roles were sufficiently represented in the research cohort. The median number of critical issues experienced by academic research staff was three. Among these, the three most frequently reported were related to: "Access to libraries / laboratories / research sites" (21.9%), "Limitation to stay abroad / study / research periods" (17.6%), "Progress of experimental work" (14.7%), with variable prevalence according to academic position and gender. Older subjects reported issues with "Projects’ financial reporting” and “Expiration of acquired consumable material more frequently”. The most common critical aspects reported in relation to the economic burden were: being “Unable to allocate funds” (31.4%), a “Reduction in clinical and scientific activity” (26.3%) and experiencing “Increased expenses (comprising private costs)” (21.2%) with no differences among genders. Researchers in Applied Sciences and Natural Sciences reported a higher frequency of problems in clinical and scientific activities, whereas increased expenses were reported also by researchers operating in the Humanities field. As a possible solution aimed at improving these critical issues, most subjects, especially those aged >45 years, indicated “Economic aid” (22.6%), “Reduction in bureaucracy” (19.9%) or “Enhancement of the scientific and clinical activities”, whereas those ≤45 years felt that an increased duration of and better access to PhD programmes were to be prioritized.

Conclusion: Our findings highlight the most critical issues related to research activities during

COVID-19 pandemic in a large academic community. The information achieved may be useful to

46	identify researchers’ needs and to design appropriate policies aimed at preparing research 47	institutions for unexpected catastrophic events and limiting the negative impact on academic

48	research activities.

# Introduction

Since early 2020, the outbreak of COVID-19 pandemic has disrupted not only societal and individual lifestyles around the world but has also heavily affected and impaired scientific research activities. Beside causing an enormous impact on healthcare systems, the pandemic outbreak caused by SARS-CoV-2 has, also heavily limited research programs in all academic fields by interfering with key steps of research activities at multiple levels [1]. A recent publication by Myers et al. [2] reported that since the onset of the COVID-19 pandemic and the consequent disruption of research activities, faculties have lost, on average, 24% of their research productivity. Laboratory-based scientists have been the most affected, as they lost up to 40% of their productivity. Indeed, many research institutions and universities from different countries severely reduced on-site academic activities [3]. A recent survey collecting data from more than 2000 subjects (scientists working in research and/or educational institutions, government agencies, industry, or other institutions involving science, technology, engineering, and mathematics [STEM]-related fields) across 132 countries reported delays in research activities and administrative duties, as well as increased workloads from adapting to online teaching environments [3]. The COVID-19 pandemic affected scientists in all fields [4], particularly in scientific fields that require laboratory resources, living animals, and time-sensitive experiments [5]. In this setting, healthcare research experienced the greatest impact. Indeed, in the UK, preliminary estimates suggest that over 1,500 academic trainees have been re-assigned exclusively to clinical activities during the pandemic [6]. Among the multiple consequences of the COVID-19 pandemic, closure of laboratories and research institutes, staff shortages, supply chain disruptions for reagents and laboratory materials as well as funding deficits have represented significant, often insurmountable, hardships for the "physiological" flow of research [7-10]. Moreover, COVID-19 pandemic has adversely affected clinical trials, which were often delayed or deferred [3]. An impressive shift of research activities towards those focused on

COVID-19 was also recorded [11]. In terms of publishing trends, some have reported a decrease in research quality and accuracy, premature publication, and a reduction in the space given to important non-COVID work [12].

In this general setting, the temporary shutdown of social relations during the pandemic, the increased caregiving responsibility for family, and the reduction of time available to work had an especially significant effect on women, who are often more involved in domestic responsibilities and childbearing duties [13-14].

Studies suggest that the pandemic exacerbated existing gender disparities for publications and career progress [15]. Indeed, numerous studies focused on disproportionate changes in publications between men and women during the pandemic [16].

Beside a general halt to research activities, emphasis has been placed on the effects on Ph.D. students, postdoctoral fellows and junior faculty, for whom the toll of lost research-time may be greater, as they are expected to produce their academic work and achieve research results within the limited timespan of their fellowships [[17].](https://paperpile.com/c/lwGxp5/NUxl) Consequently, early-career researchers (ECR) have experienced repercussions on careers and job opportunities, especially Ph.D. students and younger researchers [17-19].

At the opposite end of the spectrum in terms of consequences of the pandemic, some potentially positive "side effects", such as an acceleration of digitalization processes, adoption of remote work and remote connections, and a higher productivity in specific fields, such as artificial intelligence [20] ought to be mentioned.

The dramatic time experienced by the scientific community during COVID-19 pandemic has revealed the most fragile aspects of academic organizations and research activities, and the lessons learned should promote actions to respond and be prepared to preserve scientific activities during challenging times and emergencies.

Therefore, to explore the impact of the pandemic on research activities and in the attempt to identify the most critical issues, we performed a survey within our academic community based in

"*Sapienza*" University of Rome, the largest European university by student number with ~117.000 students. At the time of the survey, our community included 3300 academics, 845 fixed-term researchers, and 2874 Ph.D. students, all involved in academic research activities. For its size and characteristics, Sapienza University is highly representative of a large multidisciplinary academic population, spanning through different ages, genders, and career stages and acting as an international environment that boosts international relationships and cooperation (with about 9% of international students). To our knowledge, our current work is the first paper that attempts to identify the impact of COVID-19 pandemic on research activities of an entire academic community involving all areas and disciplines.

# Methods

For the scope of our research, the Governance Research Team of the University developed a questionnaire, consisting of both open- and closed-ended questions, aimed at exploring the differences in impact of the pandemic on different academic subgroups, on whom, based on the available literature, a higher toll from the pandemic might be expected. Closed-ended questions were related to the research field, the main factors influencing research activity during the COVID19 pandemic, and the consequent financial impact of research activity limitations. Open-ended questions addressed the specific effects of the financial issues on research activity and required suggestions and feedback about emerging post-pandemic research priorities. The full questionnaire is attached as Supplementary Material 1. This questionnaire, proposed in Italian to participants, comprised the following items: age, sex, academic position, department, faculty, research type, main critical issues experienced during the pandemic, additional issues, absence/presence of an economic impact (and its entity), reasons underlying the economic burden (if any), and suggestions to improve academic research and performance. The questionnaire was developed using Google Forms (Google LLC, USA). The protocol for the current survey has been approved by the Sapienza

Ethics Committee for Transdisciplinary Research (CERT) (approval no. CERT protocol ID 64/2023). Each individual participating in this survey has given explicit informed consent to the use of data. All data were collected in anonymous and aggregated form. The questionnaire was sent via email in April 2021 to all faculty members (full, associate, and assistant professors), Ph.D. students and medical residents of Sapienza University, which counts 4118 individual email addresses. Five hundred and forty-four individuals replied to the questionnaire, amounting to approximately 13,2% of the total invited cohort.

The academic position was classified into the following categories: "Ph.D. students", "Fixed-term researchers", "Full-term researchers", "Associate professors" and "Full professors".

We categorised all respondents according to their Department and Faculty into the following

Academic fields: Healthcare Professions and Applied Sciences, Humanities, Natural Sciences, Social Sciences and Formal Sciences. Regarding research type, the respondents could choose one among the following: Theoretical, Basic, Technical/Applied, Field, Clinical or Biological. Respondents were asked to specify the main and any other relevant issues affecting academic research during the pandemic, choosing within a list of 14 pre-specified possible answers. With regard to subjects experiencing an economic burden, the respondents could classify it according to the following classes: <10.000 euro, 10.000-30.000 euro, 30.000 – 60.000 euro, 60.000 – 100.000 euro and > 100.000 euro. Subjects were also asked to define the main reason underlying the experienced economic burden and to provide suggestions to restart academic research; these were open-ended questions, categorised into broad groups**.**

Responses were exported in an Excel database (Microsoft Corp, USA) and analysed using statistical software [SPSS Statistics for Windows (version 27, IBM Corp)].

Data distribution was visually inspected by analysing the respective histograms and normality plots. Data are presented as counts, percentages (%), means and standard deviations (SD), and medians with 25-75% interquartile ranges (IQR), as appropriate. Analyses were conducted employing_2_tests for categorical variables, ANOVAs, linear regression and logistic regression analyses. Given the exploratory nature of the study focused on an objective, rather than on pre-specified hypotheses, we chose not to conduct post-hoc tests or to adjust for multiple comparisons [21-23]. A robust approach using bootstrapping for 2000 samples was employed to account for non-normal data distribution, and bias-corrected accelerated (BCa) 95% confidence intervals were calculated and reported. The linear regression analyses used Stein's formula for adjusted R squared (Adj R^2^) to evaluate how well the models cross-validate across different samples of data from the same population [12], while for logistic regression analyses, we reported R^2^ values according to Nagelkerke (R^2^_N_).

The level of statistical significance was set at a P-value <0.05. All statistical computations were conducted with the IBM SPSS Statistics for Windows (version 27, IBM Corp.). The full anonymized dataset underlying the findings described in this manuscript has been uploaded in Figshare (Figshare LLC, USA). DOI: 10.6084/m9.figshare.23551743

# Results

Among the recipients of the online questionnaire-based interview, 544 responses were collected. The descriptive characteristics of the research cohort are presented in Table 1 according to the academic position, and divided on the basis of the academic field, research type and economic burden experienced. Overall, 52,2% of the respondents were female and the median age was 48 years (35-57); all academic roles were adequately represented in the research cohort, thus reflecting a substantially well-balanced sample of Ph.D. students (22.2%), Fixed-term researchers (13.1%), Full-term researchers (13.1%), Associate professors (36.0%) and Full professors (15.6%).

176

177	**Table 1.** Description of the research cohort.

|  | **Total** | **PhD student** | **Fixed-term researcher** | **Full-term researcher** | **Associate professor** | **Full professor** |
| --- | --- | --- | --- | --- | --- | --- |
| n (%) | 544 | 121 (22,2) | 71 (13.1) | 71 (13.1) | 196 (36.0) | 85 (15.6) |
| Females (%) | 52.2 | 62.8 | 62 | 54.9 | 46.4 | 40 |
| Age (yrs, [25-75% IQR]) | 48 [35-57] | 28 [27-31] | 37.5 [34-44] | 49 [43-56] | 53 [47-59] | 59 [55-63] |
| **Academic field (%)** |  |  |  |  |  |  |
| Professions and app. sciences | 33.1 | 32.2 | 36.6 | 42.3 | 30.6 | 29.4 |
| Humanities | 22.8 | 24 | 15.5 | 12.7 | 22.4 | 17.6 |
| Natural sciences | 18.4 | 18.2 | 19.7 | 16.9 | 20.4 | 14.1 |
| Social sciences | 17.6 | 17.4 | 22.5 | 25.4 | 20.4 | 20 |
| Formal sciences | 8.1 | 8.3 | 5.6 | 2.8 | 6.1 | 18.8 |
| **Research type (%)** |  |  |  |  |  |  |
| Theoretical | 30.1 | 39.7 | 26.8 | 23.9 | 26 | 34.1 |
| Basic | 20.6 | 11.6 | 23.9 | 19.7 | 28.1 | 14.1 |
| Technical/Applied | 17.5 | 19 | 21.1 | 11.3 | 15.8 | 21.2 |
| Field | 13.2 | 10.7 | 9.9 | 16.9 | 15.3 | 11.8 |
| Clinical | 11.2 | 13.2 | 8.5 | 18.3 | 8.7 | 10.6 |
| Biological | 7.4 | 5.8 | 9.9 | 9.9 | 6.1 | 8.2 |
| **Economic burden (%)** |  |  |  |  |  |  |
| None | 68 | 67.8 | 76.1 | 69 | 68.9 | 58.8 |
| <10 000 euro | 11 | 14 | 8.5 | 14.1 | 10.7 | 7.1 |
| 10 000 - 30 000 euro | 7.9 | 1.7 | 2.8 | 5.6 | 10.2 | 17.6 |
| 30 000 - 60 000 euro | 1.8 | / | 2.8 | 1.4 | 1.5 | 4.7 |
| 60 000 - 100 000 euro | 0.6 | / | 1.4 | 1.4 | 0.5 | / |
| >100 000 euro | 0.4 | / | / | / | / | 2.4 |

178

179

180

181

182

Note: academic fields were categorized into the 5 major scientific branches, **Formal sciences** (mathematics, logic, statistics, computer science, etc.), **Natural sciences** (physics, chemistry, biology, earth sciences, etc.) and **Social sciences** (archaeology, geography, anthropology, psychology, sociology, economics, political science, cultural/ethnic studies, gender studies, etc.), and **Humanities** (literature, philosophy, theology, linguistics, human history, arts, etc.) and **Professions and applied sciences** (medicine, law, agriculture, education, physical performance, consumer science, journalism, military sciences, engineering, architecture and design, transportation, business, etc.).

## Research-related issues experienced

The respondents were asked to state the main issue affecting academic research experienced during the pandemic and to list all issues encountered. The description of the main and the general research-related issues is presented in Table 2.

**Table 2.** Description of the main and overall research-related issues experienced.

| Median number of issues experienced (IQR 25-75%) | 3 [2-4] |  |
| --- | --- | --- |
| List of issues experienced (n, %) | Main | Overall |
| Access to libraries / laboratories / research sites | 119 (21.9) | 264 (48.5) |
| Limitation to abroad stay /study / research periods | 96 (17.6) | 279 (51.3) |
| Advancement of experimental work | 80 (14.7) | 183 (33.6) |
| Scientific production | 63 (11.6) | 207 (38.1) |
| Family organization | 43 (7.9) | 144 (26.5) |
| Patients’ enrollment / access | 35 (6.4) | 83 (15.3) |
| Sources retrieval | 35 (6.4) | 130 (23.9) |
| Start of experimental work / obtainment of authorizations | 24 (4.4) | 87 (16) |
| Transport logistics | 14 (2.6) | 97 (17.8) |
| Limitation to supply of services (maintenance, etc.) | 14 (2.6) | 73 (13.4) |
| Contract expiry of personnel specifically recruited for research | 8 (1.5) | 50 (9.2) |
| Projects’ financial reporting | 6 (1.1) | 63 (11.6) |
| Access to calls with deadlines (awards, grants, etc.) | 5 (0.9) | 38 (7) |
| Expiry of acquired consumable material | 2 (0.4) | 34 (6.3) |

189

The median number of issues experienced was 3 (2-4). Among the main issues, the five most frequently encountered were related to: "Access to libraries / laboratories / research site" (21.9%), "Limitation to abroad stay / study / research periods" (17.6%), "Advancement of experimental work" (14.7%), "Scientific production" (11.6%) and "Family organisation" (7.9%). Interestingly, considering all research issues experienced, these five also represented the five most frequently encountered, albeit with a different frequency: "Limitation to abroad stay / study / research periods" (51.3%), "Access to libraries / laboratories / research sites" (48.5%), "Scientific production" (38.1%), "Advancement of experimental work" (33.6%) and "Family organisation" (26.5%).

Table 3 presents the frequency of research-related issues encountered according to their academic position. Specifically, "Access to libraries / laboratories / research sites" showed a significant difference in frequency, being highest among Ph.D. students (66.9%) and Fixed-term researchers

(50.7%), whereas a "Limitation to abroad stay / study / research periods" was reported especially by

Full professors (62.4%) and Ph.D. students (62%) and "Family organisation" was frequent among Associate professors (34.2%) and Full-term researchers (28.2%). On the other hand, no significant differences were observed regarding the frequency distribution of "Advancement of experimental work" and "Scientific production".

206 **Table 3.** Research issues experienced based on academic position

**PhD students**

**Fixed**

**-**

**term**

**researchers**

**Full**

**-**

**term**

**researchers**

**Associate**

**professors**

**Full**

**professors**

***p***

**n**

121

71

71

196

85

**List of overall issues experienced [n (%)]**

| Access to libraries / laboratories / research site | 81 (66.9) | 36 (50.7) | 28 (39.4) | 78 (39.8) | 41 (48.2) | **<0.0001** |
| --- | --- | --- | --- | --- | --- | --- |
| Limitation to abroad stay /study / research periods | 75 (62) | 33 (46.5) | 20 (28.2) | 98 (50) | 53 (62.4) | **<0.0001** |
| Advancement of experimental work | 42 (34,7) | 24 (33.8) | 23 (32.4) | 65 (33.2) | 29 (34.1) | 0.99 |
| Scientific production | 44 (36.4) | 31 (43.7) | 30 (42.3) | 80 (40.8) | 22 (25.9) | 0.11 |
| Family organization | 29 (24) | 17 (23.9) | 20 (28.2) | 68 (34.2) | 11 (12.9) | **0.005** |
| Patients’ enrolment / access | 16 (13.2) | 13 (18.3) | 15 (21.1) | 27 (13.8) | 12 (14.1) | 0.53 |
| Sources retrieval | 43 (35.5) | 20 (28.2) | 13 (18.3) | 41 (20.9) | 13 (15.3) | **0.004** |
| Start of experimental work / obtainment of authorizations | 31 (25.6) | 13 (18.3) | 9 (12.7) | 26 (13.3) | 8 (9.4) | **0.011** |
| Transport logistics | 24 (19.8) | 15 (21.1) | 14 (19.7) | 33 (16.8) | 11 (12.9) | 0.64 |
| Limitation to supply of services (maintenance, etc.) | 12 (9.9) | 8 (11.3) | 10 (14.1) | 26 (13.3) | 17 (20) | 0.31 |
| Contract expiry of personnel specifically recruited for research | / | 9 (12.7) | 6 (8.5) | 19 (9.7) | 16 (18.8) | **<0.001** |
| Projects’ financial reporting | 4 (3.3) | 12 (16.9) | 6 (8.5) | 28 (14.3) | 13 (15.3) | **0.01** |
| Access to calls with deadlines (awards, grants, etc.) | 7 (5.8) | 8 (11.3) | 5 (7) | 12 (6.1) | 6 (7.1) | 0.65 |
| Expiry of acquired consumable material | 3 (2.5) | 3 (4.2) | 5 (7) | 17 (8.7) | 6 (7.1) | 0.24 |

207 Note: significant *P*-values are presented in bold.

26

208 We further analysed the impact of gender, age and academic field on the reported research issues.
209 The full data are reported in Table 4.

210

**Table 4.** Research issues experienced based on sex, age (≤45/>45 years) and scientific field

|  | **Males** | **Females** | ***p*** | **≤45 yrs** | **>45yrs** | ***p*** | **PAS** | **Hum** | **NS** | **Soc. Sci.** | **For. Sci.** | ***p*** |
| --- | --- | --- | --- | --- | --- | --- | --- | --- | --- | --- | --- | --- |
| **n** | 260 | 284 |  | 241 | 303 |  | 180 | 108 | 100 | 112 | 44 |  |
| **List of overall issues experienced (%)** |  |  |  |  |  |  |  |  |  |  |  |  |
| Access to libraries / laboratories / research site | 48.1 | 48.9 | 0.84 | 55.2 | 43.2 | **0.01** | 38.3 | 88.9 | 35 | 49.1 | 20.5 | **<0.001** |
| Limitation to abroad stay /study / research periods | 49.2 | 53.2 | 0.36 | 53.1 | 49.8 | 0.45 | 41.1 | 69.4 | 50 | 49.1 | 56.8 | **<0.001** |
| Advancement of experimental work | 33.5 | 33.8 | 0.91 | 32.4 | 34.7 | 0.58 | 43.3 | 13 | 55 | 20.5 | 29.5 | **<0.001** |
| Start of experimental work / obtainment of authorizations | 16.9 | 15.1 | 0.55 | 20.3 | 12.5 | **0.01** | 22.8 | 5.6 | 20 | 16.1 | 4.5 | **<0.001** |
| Scientific production | 39.2 | 37 | 0.60 | 41.9 | 35 | 0.10 | 37.8 | 34.3 | 40 | 33 | 56.8 | 0.07 |
| Family organisation | 25.8 | 27.1 | 0.76 | 31.5 | 22.4 | **0.02** | 20 | 26.9 | 23 | 33 | 43.2 | **0.01** |
| Patients’ enrollment / access | 10.8 | 19.4 | **0.01** | 14.5 | 15.8 | 0.67 | 29.4 | 1.9 | 6 | 19.6 | / | **<0.001** |
| Sources retrieval | 23.8 | 23.9 | 0.98 | 27.4 | 21.1 | 0.09 | 14.4 | 49.1 | 5 | 38.4 | 6.8 | **<0.001** |
| Transport logistics | 20.4 | 15.5 | 0.13 | 20.7 | 15.5 | 0.11 | 15 | 19.4 | 29 | 11.6 | 15.9 | **0.012** |
| Limitation to supply of services (maintenance, etc.) | 11.9 | 14.8 | 0.35 | 10.8 | 15.5 | 0.11 | 12.2 | 5.6 | 30 | 8.9 | 11.4 | **<0.001** |
| Contract expiry of personnel specifically recruited for research | 7.7 | 10.6 | 0.26 | 7.1 | 10.9 | 0.12 | 8.9 | 5.6 | 14 | 10.7 | 4.5 | 0.20 |
| Projects’ financial reporting | 11.5 | 11.6 | 0.99 | 8.3 | 14.2 | **0.03** | 10 | 10.2 | 11 | 17 | 9.1 | 0.39 |
| Access to calls with deadlines (awards, grants, etc.) | 5.8 | 8.1 | 0.30 | 7.9 | 6.3 | 0.46 | 6.1 | 6.5 | 5 | 9.8 | 9.1 | 0.64 |
| Expiry of acquired consumable material | 5.8 | 6.7 | 0.68 | 3.7 | 8.3 | **0.03** | 9.4 | 1.9 | 7 | 4.5 | 6.8 | 0.12 |

Note: significant *P*-values are presented in bold. Academic fields were categorised into the 5 major scientific branches, **Formal sciences** (mathematics, logic, statistics, computer science, etc.), **Natural sciences** (physics, chemistry, biology, earth sciences, etc.) and **Social sciences** (archaeology, geography, anthropology, psychology, sociology, economics, political science, cultural/ethnic studies, gender studies, etc.), and **Humanities** (literature, philosophy, theology, linguistics, human history, arts, etc.) and **Professions and applied sciences** (medicine, law, agriculture, education, physical performance, consumer science, journalism, military sciences, engineering, architecture and design, transportation, business, etc.).

Abbreviations: ***PAS* =** Professions and Applied Sciences; ***Hum*** *=* Humanities; ***NS*** = Natural Sciences; ***Soc. Sci. =*** Social Sciences; ***For. Sci.*** = Formal sciences.

26

217 No significant differences were found regarding males vs. females in the reported issues, except for 218 "Patients’' enrolment / access" which was more frequent among females.

219 We divided the research cohort according to age into two groups, ≤45 (n = 241) vs >45 years of age
220 (n = 303). The younger cohort experienced significantly more difficulties with regard to "Access to
221 libraries / laboratories / research sites", "Start of experimental work / obtainment of authorizations" 222 and "Family organisation". In contrast, the older cohort had higher rates of issues with "Projects’
223 financial reporting” and “Expiry of acquired consumable material”.
224 Most research issues significantly differed in frequency among academic fields, complete results 225 are shown in Table 4.
226 A multiple regression analysis of the number of research-related issues experienced, considering
227 respondents’ age, gender, and academic position, found a younger age as the only significant 228 independent predictor of a larger sum of overall issues reported (Table 5).

229

230

231 **Table 5.** Linear model of predictors of the number of research-related issues experienced.

***b***

**SE**

***β***

***p***

**Research-related issues number**

| Constant |  | 3.82 (3.31, 4.42) |  | 0.30 |  |  |  | <0.001 |
| --- | --- | --- | --- | --- | --- | --- | --- | --- |
| Age, years |  | -0.022 (-0.04, -0.01) |  | 0.01 |  | -0.21 |  | **0.008** |
| Sex, male |  | 0.11 (-0.12, 0.34) |  | 0.12 |  | 0.04 |  | 0.35 |
| Academic position |  | -0.05 (-0.04, -0.15) |  | 0.05 |  | 0.09 |  | 0.25 |

232 *Note:* 95% bias corrected accelerated (BCa) confidence intervals, SE and *P* values from 2000 bootstrapped samples are reported in 233 parentheses. Significant *P*-values are presented in bold. *P* for the model = 0.006; Adj R^2^ = 0.01.

234	Abbreviations: ***β*** = standardized coefficient; ***b*** = unstandardized coefficient; **SE =** standard error.

## Experience and causes of Economic burden

Overall, 117 respondents (21.7%) reported the presence of an economic burden deriving from the pandemic, with no significant differences according to gender, age group or academic field. In half of the cases reporting an economic burden, it was below 10 000 euros. Complete results are presented in Table 6.

26

240 **Table 6.** Presence of economic burden, causes and suggestions to improve research according to sex, age range (45/>45 years) and scientific field.

|  | **Total** | **Males** | **Females** | ***p*** | **≤45yrs** | **>45yrs** | ***p*** | **PAS** | **Hum** | **NS** | **Soc. Sci.** | **For. Sci.** | ***p*** |
| --- | --- | --- | --- | --- | --- | --- | --- | --- | --- | --- | --- | --- | --- |
| **n** | 544 | 260 | 284 |  | 241 | 303 |  |  |  |  | 96 | 44 |  |
| **Presence of any economic burden (%)** | 21.7 | 25 | 18.7 | 0.07 | 19.5 | 23.4 | 0.27 |  |  |  | 13.5 | 13.6 | 0.13 |
| **List of reasons for economic burden (%)** |  |  |  |  |  |  |  |  |  |  |  |  |  |
| Unable to allocate funds | 31.4 | 27.7 | 35.8 | 0.19 | 28.6 | 38.8 | 0.28 | 30.2 | 28 | 47.8 | 33.3 | 50 | 0.52 |
| Reduction in clinical and scientific activity | 26.3 | 30.8 | 20.8 | 0.19 | 19 | 35.8 | 0.06 | 39.5 | 8 | 39.1 | 16.7 | 33.3 | **0.046** |
| Increased expenses (comprising private costs) | 21.2 | 21.5 | 20.8 | 0.99 | 31 | 17.9 | 0.12 | 27.9 | 40 | / | 25 | / | **0.01** |
| Interrupted mobility programmes | 5.9 | 6.2 | 5.7 | 0.95 | 9.5 | 4.5 | 0.30 | 2.3 | 8 | 4.3 | 25 | / | 0.07 |
| Reduction in the number of calls and grants available | 2.5 | 3.1 | 1.9 | 0.71 | 2.4 | 3 | 0.85 | / | 8 | / | / | 16.7 | 0.06 |
| Closure of archives and libraries | 1.7 | 1.5 | 1.9 | 0.86 | 4.8 | / | 0.07 | / | 8 | / | / | / | 0.14 |
| Lack of personnel | 1.7 | 1.5 | 1.9 | 0.86 | 4.8 | / | 0.07 | / | / | 8.7 | / | / | 0.11 |
| **Suggestions to improve academic research (%)** |  |  |  |  |  |  |  |  |  |  |  |  |  |
| Economic aid | 22.6 | 19.4 | 25.2 | 0.24 | 18.6 | 25.1 | 0.19 | 23 | 13.2 | 38.5 | 24 | 10.5 | **0.01** |
| Reduction in bureaucracy | 19.9 | 23.9 | 16.6 | 0.12 | 12.7 | 24.6 | **0.012** | 19 | 15.8 | 21.2 | 18 | 42.1 | 0.14 |
| Enhancement of the scientific and clinical activities | 19.2 | 20.1 | 18.4 | 0.71 | 18.6 | 19.6 | 0.85 | 25 | 11.8 | 13.5 | 24 | 21.1 | 0.15 |
| Increasing the PhD length and ease of access | 11.1 | 8.2 | 13.5 | 0.15 | 22.9 | 3.4 | **<0.00**    **1** | 14 | 6.6 | 11.5 | 14 | 5.3 | 0.48 |
| Digitalization | 9.8 | 11.9 | 8 | 0.25 | 8.5 | 10.6 | 0.54 | 7 | 22.4 | 3.8 | 6 | / | **0.001** |
| Resumption of presence activities | 6.4 | 6.7 | 6.1 | 0.84 | 3.4 | 8.4 | 0.09 | 4 | 6.6 | 7.7 | 6 | 15.8 | 0.42 |
| Re-opening of archives and libraries | 5.7 | 5.2 | 6.1 | 0.74 | 10.2 | 2.8 | **0.008** | 2 | 15.8 | / | 6 | / | **<0.00**  **1** |
| Increasing the personnel | 3 | 2.2 | 3.7 | 0.47 | 2.5 | 3.4 | 0.69 | 5 | 3.9 | 1.9 | / | / | 0.42 |
| Enhancement of abroad mobility programmes | 2.4 | 2.2 | 2.5 | 0.90 | 2.5 | 2.2 | 0.86 | 1 | 3.9 | 1.9 | 2 | 5.3 | 0.66 |

241 Note: significant *P*-values are presented in bold. Academic fields were categorised into the 5 major scientific branches, **Formal sciences** (mathematics, logic, statistics, computer science, etc.), **Natural sciences** 242 (physics, chemistry, biology, earth sciences, etc.) and **Social sciences** (archaeology, geography, anthropology, psychology, sociology, economics, political science, cultural/ethnic studies, gender studies, etc.), and 243 **Humanities** (literature, philosophy, theology, linguistics, human history, arts, etc.) and **Professions and applied sciences** (medicine, law, agriculture, education, physical performance, consumer science, 244 journalism, military sciences, engineering, architecture and design, transportation, business, etc.).
245 Abbreviations: ***PAS* =** Professions and Applied Sciences; ***Hum*** *=* Humanities; ***NS*** = Natural Sciences; ***Soc. Sci. =*** Social Sciences; ***For. Sci.*** = Formal sciences.

26

246 The three most frequently reported reasons underlying an economic burden were: being “Unable to
247 allocate funds” (31.4%), a “Reduction in clinical and scientific activity” (26.3%) and experiencing
248 “Increased expenses (comprising private costs)” (21.2%). No specific differences were reported
249 between males and females or between younger and older respondents. However, those in the fields 250 of Healthcare Professions and Applied Sciences and Natural Sciences reported a higher frequency 251 of problems in clinical and scientific activities (39.5% and 39.1%, respectively)~~,~~ while increased 252 (also personal) expenses were reported by those in the Humanities field (40%).
253 When analysed according to academic role, Ph.D. students reported “Increased expenses
254 (comprising private costs)” more frequently when compared to other groups (38.9%), and they were
255 the only category to mention “Closure of archives and libraries” as problematic (11.1%). Full 256 results are reported in Table 7.

26

257 **Table 7.** Presence of economic burden, causes and suggestions to improve research according to academic position.

|  | **PhD student** | **Fixed-term researcher** | **Full-term researcher** | **Associate professor** | **Full professor** | ***p*** |
| --- | --- | --- | --- | --- | --- | --- |
| **n** | 121 | 71 | 71 | 196 | 85 |  |
| **Presence of any economic burden (%)** | 15.7 | 15,5 | 22,5 | 23 | 31.8 | 0.05 |
| **List of reasons for economic burden (%)** |  |  |  |  |  |  |
| Unable to allocate funds | 22.2 | 60 | 26.7 | 33.3 | 40.7 | 0.30 |
| Reduction in clinical and scientific activity | 16.7 | 20 | 20 | 30.8 | 44.4 | 0.25 |
| Increased expenses (comprising private costs) | 38.9 | 10 | 40 | 23.1 | 7.4 | **0.044** |
| Interrupted mobility programmes | 11.1 | / | / | 10.3 | 3.7 | 0.47 |
| Reduction in the number of calls and grants available | / | 10 | 6.7 | / | 3.7 | 0.35 |
| Closure of archives and libraries | 11.1 | / | / | / | / | **0.034** |
| Lack of personnel | / | / | 6.7 | 2.6 | / | 0.56 |
| **Suggestions to improve academic research (%)** |  |  |  |  |  |  |
| Economic aid | 16.4 | 25 | 25.7 | 23.5 | 24.6 | 0.75 |
| Reduction in bureaucracy | 10.4 | 5.6 | 20 | 29.4 | 22.8 | **0.005** |
| Enhancement of the scientific and clinical activities | 17.9 | 19.4 | 14.3 | 18.6 | 24.6 | 0.80 |
| Increasing the PhD length and ease of access | 31.3 | 11.1 | 2.9 | 5.9 | 1.8 | **<0.001** |
| Digitalization | 4.5 | 13.9 | 17.1 | 9.8 | 8.8 | 0.29 |
| Resumption of presence activities | 4.5 | 5.6 | 8.6 | 5.9 | 8.8 | 0.86 |
| Re-opening of archives and libraries | 10.4 | 11.1 | 2.9 | 2.9 | 3.5 | 0.13 |
| Increasing the personnel | 1.5 | 8.3 | 5.7 | 2 | 1.8 | 0.24 |
| Enhancement of abroad mobility programmes | 3 | / | 2.9 | 2 | 3.5 | 0.84 |

258 Note: significant *P*-values are presented in bold. Academic fields were categorized into the 3 major scientific branches, **Formal sciences** (mathematics, logic, statistics, computer science, etc.), **Natural** 259 **sciences** (physics, chemistry, biology, earth sciences, etc.) and **Social sciences** (archaeology, geography, anthropology, psychology, sociology, economics, political science, cultural/ethnic studies, gender 260 studies, etc.), and **Humanities** (literature, philosophy, theology, linguistics, human history, arts, etc.) and **Professions and applied sciences** (medicine, law, agriculture, education, physical performance, 261 consumer science, journalism, military sciences, engineering, architecture and design, transportation, business, etc.).

262	Abbreviations: ***PAS* =** Professions and Applied Sciences; ***Hum*** *=* Humanities; ***NS*** = Natural Sciences; ***Soc. Sci. =*** Social Sciences; ***For. Sci.*** = Formal sciences.

26

In a logistic regression analysis on the presence of economic burden, comprising age, gender and academic role, only the position of Full professor was found to be a significant independent predictor of the presence of (any) economic burden **(**Table 8**)**.

**Table 8.** Logistic regression analysis of the presence of economic burden.

***b***

**SE**

***β***

***p***

**Presence**

**of economic burden**

Constant

-

0.93 (

-

1.99

,

0.03)

0.56

0.09

| Age, years |  | -0.02 (-0.05, 0.01) |  | 0.02 |  | 0.98 |  | 0.21 |
| --- | --- | --- | --- | --- | --- | --- | --- | --- |
| Sex, male |  | -0.30 (-0.74, 0.16) |  | 0.23 |  | 0.74 |  | 0.18 |
| Fixed-term researcher |  | 0.17 (-0.82, 1.04) |  | 0.47 |  | 1.18 |  | 0.70 |
| Full-term researcher |  | 0.78 (-0.26, 1.81) |  | 0.52 |  | 2.19 |  | 0.11 |
| Associate professor |  | 0.87 (-0.13, 1.91) |  | 0.47 |  | 2.38 |  | 0.07 |
| Full professor |  | 1.36 (0.11, 2.62) |  | 0.57 |  | 3.89 |  | **0.016** |

*Note:* 95% bias corrected accelerated (BCa) confidence intervals, SE and *P* values from 2000 bootstrapped samples are reported in parentheses. Significant *P*-values are presented in bold. *P* for the model = 0.06; R^2^_N_ = 0.034.

Abbreviations:	***β***	=	standardized	coefficient;	***b***	=	unstandardized	coefficient;	**SE	=**	standard	erro

26

## Suggestions to support academic research

Respondents were asked to provide suggestions on how to improve and “recover” academic research status; answers were categorised into nine broad groups and the results are reported in Table 6.

The three most frequent suggestions were the need of an “Economic aid” (22.6%), asking for a

“Reduction in bureaucracy” (19.9%), or the “Enhancement of the scientific and clinical activities” (19.2%).

No significant differences were reported according to gender. Those ≤45 years of age felt that increasing the length and improving the ease of access of the Ph.D. programme (22.9% vs 3.4%) was more necessary, together with re-opening archives and libraries (10.2% vs. 2.8%), while those >45 years of age suggested a reduction in bureaucracy more frequently (24.6% vs 12.7%).

Among the different academic fields, Natural Sciences requested more “Economic aid” while Humanities favoured a larger adoption of “Digitalization” and a prompt “Re-opening of archives

and libraries”.

A “Reduction in bureaucracy” was advocated especially by Associate and Full professors (29.4% and 22.8%, respectively), and unsurprisingly, PhD students suggested “Increasing the PhD length and ease of access” (31.3%). The full results are reported in Table 6.

# Discussion

The global storm that hit human activities during COVID-19 pandemic has heavily and broadly affected academic research activities. Although several previous reports have proposed analyses mostly focused on the hardships in specific fields, such as science and medicine, other important aspects have been substantially neglected. A comprehensive exploratory survey, encompassing research difficulties in different disciplines and at different career stages, was lacking in Europe.

For this reason, we investigated a representative sample of the academic population of “*Sapienza*” University with a web-based questionnaire. This study presents a unique, qualitative data-driven representation of the impact of COVID-19 on academic research activities in Italy. The results of our survey highlight several critical issues that influenced research activities and could contribute to develop future strategies to limit the heavy consequences of pandemics or other catastrophic events in the future, both in Italy and in other European countries with similar academic systems.

A first conclusion that can be drawn from the analysis of our survey is that most of the pre-existing drawbacks and barriers to academic activity have been amplified during the COVID-19 pandemic. In fact, the responses to the questionnaires reflected several weaknesses exacerbated by the pandemic which may require attention and preventive measures. Respondents reported a median of 3 research-related critical issues, with no significant differences among gender, age groups, academic position, research field or type. However, when accounting for age, gender and academic position, younger age was independently associated with a higher number of experienced critical issues (Table 5**)**. When asked about the main issue impacting research, difficulties concerning impaired access to research facilities (such as laboratories, libraries and research sites) emerged as the most frequent (21.9%) among respondents. This issue, although common to many (overall 48.5%) and in all academic fields, was significantly more prevalent among Ph.D. students (66.9%), younger researchers (55.2%) and extremely frequent in the field of Humanities (88.9%). This issue has been widely recognized as a main problem to research activities, especially scientific, during the pandemic. Often the typical worksite has been replaced by working at home in inadequate environmental conditions for the younger populations [24]. The impact on clinical science was also significantly affected due to many clinical trials having been paused or terminated due to pandemic restrictions, quarantines and lockdowns; similarly, patient enrolment for new studies has been frequently halted. These circumstances have prompted a specific set of publications from the US *Food and Drug Administration [U.S. Department of Health and Human Services, Food and Drug Administration. FDA guidance on conduct of clinical trials of medical products during COVID-19 public health emergency: guidance for industry, investigators, and institutional review boards. https://www.fda.gov/media/136238/download]* and from the European Medical Agency *[Guidance on the management of clinical trials during the COVID-19 (Coronavirus) pandemic*

[*https://ec.europa.eu/health/system/files/2021-02/guidanceclinicaltrials_covid19_en_0.pdf]*](https://ec.europa.eu/health/system/files/2021-02/guidanceclinicaltrials_covid19_en_0.pdf) guidelines on the conduct of clinical trials during the COVID-19 pandemic to preserve trial integrity, to ensure compliance and to assure subjects’ and patients’ safety.

On the other hand, when considering all problems encountered, travel limitations restricting abroad stay, study and/or research periods were felt as the most frequent issue overall (51.3%), despite having been raised as the main issue by only 17.6% of respondents. Ph.D. students, alongside full professors, reported this issue the most (62 and 62.4%, respectively), with the highest rates in the fields of Humanities (69.4%) and Formal Sciences (56.8%). This limitation has also affected scientific exchange, which has produced a global shift of many international conferences into virtual platforms for teaching and knowledge dissemination, thus somewhat limiting the opportunities for networking [17] and producing effects and “technostress” burnout [18]. A decrease in scientific contacts and networks during the pandemic, with subsequent impaired opportunities for interactions and peer support, has been reported [11]. On the other hand, global scientific collaboration has reached unprecedented levels, owing to data sharing about COVID-19 related analyses. Although fruitful, such endeavours have raised numerous issues including how to uphold the basic standards of scientific conduct and integrity [12]. Our survey also focused on the incidence of pandemic-associated economic burden on academics. Some degree of burden was reported approximately by 1 out of 5 respondents, with no significant differences according to gender, age or academic field. Inability to allocate research funds and a reduction in clinical activities were the most common reasons behind this burden. Indeed, with the prioritisation of COVID-19 studies, new clinical trials have been paused, with detrimental impacts on clinical academics involved in non-COVID 19 research [25]. However, increased expenses, also accounting for private costs, were also significantly present among Ph.D. students and researchers, especially in the field of Humanities. In a multiple logistic regression analysis to identify the predictors of an economic burden only the role of Full professor was independently associated with (any) economic burden, possibly because of the larger amount of funds managed. Part of our findings are in accordance with published literature, which reports a higher impact on ECR and on “wet laboratory researchers” [26-27]. As PhD students’ and fixed-term researchers’ positions are usually based on fellowship programmes lasting a limited amount of time, the lost (research) time has reasonably determined a heavy toll on these researchers, as they are expected to produce their scientific work and reach their research objectives within a limited time span [28]. Consequently, this academic population is particularly vulnerable to the impact of these conditions on their research output, because of delays in publication, partly deriving from difficulties in completing experimental work.

All these factors may reduce smaller opportunities for ECR’s funding applications and for job applications, in the current context of job cuts and hiring freeze due to global financial pressures. On the other hand, many journals have granted deadline extensions for submitting academic work, financial reporting of ongoing projects has been postponed by some research institutions and universities, and the time limit for the expense of funding derived from grants has often been extended. Wet researchers were particularly affected from the closure of laboratories and research institutions, as the loss of experimental work has been estimated to be between 1 and 6 months in approximately 25% of cases [2], compared to “dry research” that can be at least in part conducted remotely. Specifically, as previously reported, researchers working with cell cultures suffered widespread supply shortages concerning (e.g. qPCR) reagents and consumables (especially plasticware), but also including personal protective equipment (PPE), such as gloves and masks [3;7]. Similarly, researchers working with laboratory animals also experienced difficulties deriving from staff shortages, disruption in the supply chain of (veterinary) drugs and necessary tools for animal care and housing [2,7].

Our results partly differ from the available literature. No significant differences were found in the rates of research-related issues among male and female researchers, except for greater difficulties in women concerning patients’ enrolment and access, possibly due to a higher number of female physicians in our sample. Regarding researchers experiencing an economic burden, no significant differences were found between male and female, with a trend favouring females.

An equal rate of men and women experiencing difficulties with family organisation impacting their research activities emerged from our survey (25.8% vs 27.1%, respectively). This contrasts with published literature and reports focusing on the risks intrinsic to gender inequality in the academic context. In fact, female academics have been reported to more likely assume parenting or domestic responsibilities than their male counterparts. Furthermore, female researchers are more likely to shoulder domestic duties following the closure of universities and research institutions, as well as childbearing and home-schooling when lockdowns also involved schools and kindergartens [29].

Indeed, it has been postulated that many women are currently doing ‘second and third shifts’ regarding housework and welfare after their first shift of paid work [30]. The possible explanation of our data relies on the lack of information on family composition and the presence of offspring from participants in our survey, as parents are expected to suffer more significantly from the issues mentioned above [24]. Moreover, we do not have data about how the number of working hours has changed during the pandemic for male and female researchers. Indeed, data suggest a differential amount of available working hours for male/female and parents/not parents researchers [3]. Recent evidence suggest that gender-related issues emerged during COVID-19 have lasted well beyond the first wave. In 2020-2021 gender inequalities for women working in biology, biochemistry, and civil and environmental engineering at universities, have remained steady or worsened. Compared to men, a greater proportion of women who responded to surveys reported inability to focus on their research activities. Moreover, female scientists were also more likely to report having grant disruptions and research grants that face financial difficulties due to the pandemic. Furthermore, women make up only a third of authors named on COVID-19 related publications, and they are particularly underrepresented among first and last authorship positions [31]. An analysis on more than 2000 Elsevier journals during the first wave of the pandemic has shown that women submitted proportionally fewer manuscripts than men. This deficit was especially pronounced among junior cohorts of women academics [32]. This gender-related issue, requires more extensive analysis, since our current survey may not produce sufficient information.

As our work aimed at identifying key issues and possible resilience strategies that could help resume academic activities after the pandemic, we asked our community for suggestions. Notably, economic aid was felt necessary by 22.6%, especially by respondents in the field of Natural Sciences (38.5%). In contrast, older researchers favoured a reduction in bureaucracy in approximately a quarter of cases, Ph.D. students unsurprisingly suggested to increase the Ph.D. length and ease of access (31.3%) and Humanities researchers supported the need for a more widespread process of digitalization (22.4%) and advocated for the re-opening of archives and libraries (15.8%).

## Limitations

The present survey consisted of closed- and open-ended questions sent by email to people involved in research activities in Sapienza University. Some elements were not intended to be investigated in the survey, such as the quantification of the amount of time dedicated to research and different activities (teaching, mentoring etc) during the pandemic as well as the quality of remote work from home. Furthermore, our approach was focused on the subjective perceptions of the impact and consequences of COVID-19 on their own research, in qualitative rather than quantitative terms. Moreover, the composition of the family of respondents was not assessed, thus limiting the chance to derive conclusions about issues specifically related to parenthood. Lastly, it is unclear whether the findings and the conclusions drawn from our study may be similarly applied to other academic institutions in Italy or in Europe. However, our study has been conducted in the largest Italian University and one of the largest academic communities in Europe and its findings are largely consistent with previous similar studies.

### Conclusions

To our knowledge, this is the first thorough exploratory analysis of the impact of the COVID-19 pandemic on a population sample such as that included in our survey, which represents the whole active academic multidisciplinary research community in a large University. This provides many new potential useful insights for planning systematic changes to scientific institutions before and during new possible global crises. We had a heterogeneous study population in terms of position, rank, career stage, tenure status, discipline, and characteristics of work. Our results may represent a significant reference for policy makers, university administrators, and scientists about issues exacerbated by COVID-19 related policies and suggest a fundamental need to develop approaches future crises. Indeed, our findings, beside their potential intrinsic informative value, may be useful to design appropriate policies aimed at limiting the impact of unexpected catastrophic events on research, pointing out what could be needed to preserve and even reinforce research facilities and activities in critical situations like those caused by the SARS-COV-2 pandemic.

**Disclosures:**

Acknowledgements: The authors are grateful to the members of Sapienza Governance Research

Team: Mariasabrina Sarto, Francesca Bozzano, Carlo Catalano, Luciano Galantini, Marco Oliverio, Daniele Riccioni, Mauro Valorani, Antonella Cammisa, Ciro Franco, Andrea Riccio, Fabio Sciarrino.

Francesco Carlomagno, MD, PhD and Allegra Battistoni MD, PhD gave a substantial contribution in the development of the manuscript.

Melwyn Luis Muthukkattil, native English language speaker, has revised the manuscript.

Other disclosures: None

Ethical approval: Not applicable

Disclaimers: None

Previous presentations: None

Data sharing: no additional data

**S1 Appendix: Questionnaire in English**

#### References

1. Sohrabi C , Mathew G, Franchi T, Kerwan A, Griffin M, Del Mundo JSC et al. Impact of the coronavirus (COVID-19) pandemic on scientific research and implications for clinical academic training – A review. Int J Surg*.* 2021; 86:57–63
2. Carr RM, Lane-Fall MB, South E, Brady D, Momplaisir F, Guerra CE, et al. Academic careers and the COVID-19 pandemic: Reversing the tide. Sci Transl Med. 2021;13:eabe7189.
3. Heo S, Chan AY, Diaz Peralta P, Jin L, Pereira Nunes CR, Bell ML. Impacts of the COVID-19 pandemic on scientists' productivity in science, technology, engineering, mathematics (STEM), and medicine fields. Humanit Soc Sci Commun. 2022; 9:434
4. Myers KR, Tham WY, Yin Y, Cohodes N, Thursby JG, Thursby MC, Schiffer P, Walsh JT,

Lakhani KR, Wang D, Unequal effects of the COVID-19 pandemic on scientists. Nat. Hum.

Behav. 2020; 4: 880–883

5. Mehta N, Inamdar V, Puthillam A et al. Assessing the impact of COVID-19 on STEM (science, technology, engineering, mathematics) researchers in India. Wellcome Open Res 2020; 7:157.
6. National Institute for Health Research, Progressing UK clinical academic training in 2020: addressing the challenges of COVID-19. https://www.nihr.ac.uk/document s/progressing-ukclinical-academic-training-in-2020-addressing-the-challenges-ofcovid-19/24958, 2020
7. Korbel JO, Stegle O. Effects of the COVID-19 pandemic on life scientists. Genome Biol*.* 2020;

21:1–5.

8. Grimm D. Respirators, quarantines, and worst-case scenarios: Lab animal facilities grapple with the pandemic. Science. 2020 Mar 18; Available from:

https://www.sciencemag.org/news/2020/03/respirators-quarantines-and-worst-case-scenarioslab-animal-facilities-grapple-pandemic

9. Madhusoodanan J. Frozen cells and empty cages: researchers struggle to revive stalled experiments after the lockdown. *Nature*. 2020 Jun 5. doi: 10.1038/d41586-020-01704-y.;

Available from: <https://www.nature.com/articles/d41586-020-01704-y>

10. Rimmer A. Covid-19: How clinical academic trainees came back to the wards en masse. *BMJ*.

2021; 373:n558. doi: 10.1136/bmj.n558

11. Sohrabi C, Mathew G, Franchi T, Kerwan A, Griffin M, Soleil C Del Mundo J, Ali SA, Agha M, Agha R. Impact of the coronavirus (COVID-19) pandemic on scientific research and implications for clinical academic training - A review. Int J Surg. 2021; 86:57-63.
12. Smith EMR, Rakestraw C, Farroni JS. Research integrity during the COVID-19 pandemic:

Perspectives of health science researchers at an Academic Health Science Center. Account Res.

2022:1-22. Epub ahead of print

13. Staniscuaski F, Reichert F, Werneck FP, de Oliveira L, Mello-Carpes PB, Soletti RC, et al.

Impact of COVID-19 on academic mothers. Science. 2020; 368:724.

14. Malisch JL, Harris BN, Sherrer SM, Lewis KA, Shepherd SL, McCarthy PC, et al. Opinion: In the wake of COVID-19, academia needs new solutions to ensure gender equity. Proc Natl Acad Sci U S A. 2020; 117:15378–15381.
15. Staniscuaski F, Kmetzsch L, Soletti RC, Reichert F, Zandonà E, Ludwig ZMC,et al Gender,

Race and Parenthood Impact Academic Productivity During the COVID-19 Pandemic: From

Survey to Action. Front Psychol. 2021; 12:663252

16. Bell ML, Fong KC. Gender Differences in First and Corresponding Authorship in Public Health Research Submissions During the COVID-19 Pandemic. Am J Public Health. 2021; 111:159163.
17. Paula JR. Lockdowns due to COVID-19 threaten PhD students’ and early-career researchers’ careers. Nature Ecology & Evolution. Nat Ecol Evol*.* 2020; 4:999
18. Woolston C. Pandemic darkens postdocs’ work and career hopes. Nature. 2020; 585:309-312.
19. Woolston C. Junior researchers hit by coronavirus-triggered hiring freezes. Nature*.*

2020;582:449–450.

20. Sreepadmanabh M, Sahu AK, Chande A. COVID-19: Advances in diagnostic tools, treatment strategies, and vaccine development. J Biosci. 2020; 45:148.
21. Groenwold RHH, Goeman JJ, Cessie SL, Dekkers OM. Multiple testing: when is many too much? Eur J Endocrinol. 2021; 184: E11-E14
22. Ranstam J. Multiple P-values and Bonferroni correction. Osteoarthritis Cartilage. 2016; 24:763-

764

23. Bender R, Lange S. Adjusting for multiple testing--when and how? J Clin Epidemiol.

2001;54:343-9

24. Heo S, Chan AY, Diaz Peralta P, Jin L, Pereira Nunes CR, Bell ML. Impacts of the COVID-19 pandemic on scientists' productivity in science, technology, engineering, mathematics (STEM), and medicine fields. Humanit Soc Sci Commun. 2022; 9:434
25. Finn GM, Crampton P, Buchanan JA, Balogun AO, Tiffin PA, Morgan JE, et al. The impact of the COVID-19 pandemic on the research activity and working experience of clinical academics, with a focus on gender and ethnicity: a qualitative study in the UK. BMJ Open.

2022; 12:e057655

26. Bishr Omary M, Hassan M. Opinion: Here’s how we restore productivity and vigor to the biomedical research workforce in the midst of COVID-19*.* Proc Natl Acad Sci U S A. 2020;

117:19612–19614.

27. Byrom N. COVID-19 and the Research Community: The challenges of lockdown for earlycareer researchers. eLife. 2020; 9: e59634. Published online 2020 Jun 12. doi:

10.7554/eLife.59634

28. Termini CM, Traver D. Impact of COVID-19 on early career scientists: an optimistic guide for the future. BMC Biol. 2020; 18:95.
29. Gewin V. Pandemic burnout is rampant in academia. Nature. 2021; 591:489–491
30. Chung H. Return of the 1950s housewives? how to stop coronavirus lockdown reinforcing sexist gender roles, 2020. Available: https:// theconversation.com/return-of-the-1950s housewife-how-to-stopcoronavirus-lockdown-reinforcing-sexist-gender-roles-134851.
31. Pinho-Gomes AC, Peters S, Thompson K, Hockham C, Ripullone K, Woodward M, et al. Where are the women? Gender inequalities in COVID-19 research authorship. BMJ Glob

Health. 2020; 5:e002922

32. Squazzoni F, Bravo G, Grimaldo F, García-Costa D, Farjam M, Mehmani B. Gender gap in journal submissions and peer review during the first wave of the COVID-19 pandemic. A study on 2329 Elsevier journals. PLoS One. 2021; 16:e0257919

Response to Reviewers

Journal requirements:

When submitting your revision, we need you to address these additional requirements.

1. Please ensure that your manuscript meets PLOS ONE's style requirements, including those for file naming. The PLOS ONE style templates can be found at

[https://journals.plos.org/plosone/s/file?id=wjVg/PLOSOne_formatting_sample_main_body. pdf](https://journals.plos.org/plosone/s/file?id=wjVg/PLOSOne_formatting_sample_main_body.pdf) and

[https://journals.plos.org/plosone/s/file?id=ba62/PLOSOne_formatting_sample_title_author s_affiliations.pdf](https://journals.plos.org/plosone/s/file?id=ba62/PLOSOne_formatting_sample_title_authors_affiliations.pdf)

Answer: done

2. Please provide additional details regarding participant consent. In the ethics statement in the Methods and online submission information, please ensure that you have specified (1) whether consent was informed and (2) what type you obtained (for instance, written or verbal, and if verbal, how it was documented and witnessed). If your study included minors, state whether you obtained consent from parents or guardians. If the need for consent was waived by the ethics committee, please include this information.

If you are reporting a retrospective study of medical records or archived samples, please ensure that you have discussed whether all data were fully anonymized before you accessed them and/or whether the IRB or ethics committee waived the requirement for informed consent. If patients provided informed written consent to have data from their medical records used in research, please include this information

Once you have amended this/these statement(s) in the Methods section of the manuscript, please add the same text to the “Ethics Statement” field of the submission form (via “Edit Submission”).

For additional information about PLOS ONE ethical requirements for human subjects research, please refer to [http://journals.plos.org/plosone/s/submission-guidelines#lochuman-subjects-research.](http://journals.plos.org/plosone/s/submission-guidelines#loc-human-subjects-research)

Response: done

3. We note that you have included the phrase “data not shown” in your manuscript. Unfortunately, this does not meet our data sharing requirements. PLOS does not permit references to inaccessible data. We require that authors provide all relevant data within the paper, Supporting Information files, or in an acceptable, public repository. Please add a citation to support this phrase or upload the data that corresponds with these findings to a stable repository (such as Figshare or Dryad) and provide and URLs, DOIs, or accession numbers that may be used to access these data. Or, if the data are not a core part of the research being presented in your study, we ask that you remove the phrase that refers to these data.

Response: Following journal’s requirements, we have uploaded the full anonymized dataset underlying the findings described in this manuscript in Figshare. DOI to uploaded file is:

10.6084/m9.figshare.23551743 We have integrated the materials and methods section specifying that the full dataset has been uploaded in Figshare. Data Availability Statement: Data is available from Figshare (DOI: 10.6084/m9.figshare.23551743).

4. Check the last line of the abstract to ensure it is the same. The reason for this check is to ensure that the AEs and Reviewers are sent correct information to allow them to make a good decision on whether they can manage/review the manuscript. Only send back for a change if the abstract on EM and in the manuscript are VASTLY different.

Response: Dear Editor, the abstract has been changed.

[Note: HTML markup is below. Please do not edit.]

Reviewers' comments:

Reviewer's Responses to Questions

**Comments to the Author**

1. Is the manuscript technically sound, and do the data support the conclusions?

The manuscript must describe a technically sound piece of scientific research with data that supports the conclusions. Experiments must have been conducted rigorously, with appropriate controls, replication, and sample sizes. The conclusions must be drawn appropriately based on the data presented.

Reviewer #1: Partly

Reviewer #2: Partly

2. Has the statistical analysis been performed appropriately and rigorously?

Reviewer #1: Yes

Reviewer #2: No

3. Have the authors made all data underlying the findings in their manuscript fully available?

The [PLOS Data policy](http://www.plosone.org/static/policies.action#sharing) requires authors to make all data underlying the findings described in their manuscript fully available without restriction, with rare exception (please refer to the Data Availability Statement in the manuscript PDF file). The data should be provided as part of the manuscript or its supporting information, or deposited to a public repository. For example, in addition to summary statistics, the data points behind means, medians and variance measures should be available. If there are restrictions on publicly sharing data— e.g. participant privacy or use of data from a third party—those must be specified.

Reviewer #1: No

Reviewer #2: No

4. Is the manuscript presented in an intelligible fashion and written in standard English?

PLOS ONE does not copyedit accepted manuscripts, so the language in submitted articles must be clear, correct, and unambiguous. Any typographical or grammatical errors should be corrected at revision, so please note any specific errors here.

Reviewer #1: No

Reviewer #2: No

5. Review Comments to the Author

Please use the space provided to explain your answers to the questions above. You may also include additional comments for the author, including concerns about dual publication, research ethics, or publication ethics. (Please upload your review as an attachment if it exceeds 20,000 characters)

Reviewer #1: Abstract;

Needs some review Introduction:

The authors proposed an interesting research, however the introduction needs to some adjustments. What is the context of the study (needs improvement); explore the importance of the theme (needs improvement); the main goals and relevance of the study (good) how this study will contribute (add).

Response: We thank the reviewer for this comment. We have largely modified the introduction. In particular, we added in the introduction section paragraphs giving more insights and context in terms of existing literature, the importance of the subject matter, and the relative contribution of our study to the scientific knowledge that is currently available. Please see the red parts in the text.

L60-61 discuss better: how double/triple journey of female researcher would be impacted specific in the context of the pandemic? The sentence is very general and accounts for what is already known. Ref.: Davis, P.B., Meagher, E.A., Pomeroy, C. et al. Pandemicrelated barriers to the success of women in research: a framework for action. Nat Med 28, 436–438 (2022). <https://doi.org/10.1038/s41591-022-01692-8>

Response: We thank the reviewer for this comment. Gender-related issues in research and teaching during the pandemic have been largely addressed in the new version with the relevant references. Generally, it seems that pre-existing barriers to academic activity have become amplified during the COVID-19 pandemic. Our results are not fully consistent with some of the previous reports, probably as a consequence of some features of the information required in the questionnaires which we acknowledged as a limitation. We have therefore added a paragraph about female researchers facing the pandemic in the introduction as well as a specific discussion. Please see red parts in the text.

L62-64 what about the race to research and publish articles in health science? Investments in vaccines? Data sharing and cooperation internationally?

Response: We thank the reviewer for this comment. This is a complex issue. During the pandemic, research activities related to Covid-19 have been privileged with a consequent increase in publication of COVID-19 related papers also owing to international unpreceded data sharing. Despite this, a reduction in international cooperation and and meetings in other fields has been observed and the accuracy of fast-track COVID-19 related publications has also been questioned. We have added multiple sentences in both the introduction and discussion about this. Please see red text in the manuscript (see introduction line 71-75; discussion page 26). This aspect was not an objective of our survey although it has been extensively investigated in previous studies as we mentioned in the introduction and discussion.

Methods

Why you don't have ethical approval for this research?

How researchers developed the informed consent for participants? how you developed the questionnaires? you should at least get a weaver from the ethical committee of the university. Please contact the ethical committee of your institution and clarify this.

Response: please see method section for the ethical approval.

Results

Table 1 research type - check "filed"is it a research type? I don't understand.

Response: We are afraid the Reviewer may have misread “field (research)” as “filed (research)”. We intend Field research (or study) to comprise all subjects in which raw data is collected outside of a laboratory, workplace or library setting. This large field comprises social sciences observing people interact in their natural environment, biologists studying natural animal behaviour, and many archaeologists, anthropologists, economists, etc.

Supplementary data

make sure to add data that are relevant to the study. Check the journal policy regarding data sharing and transparency in order to be in accordance to it

Response: We thank the Reviewer for his/her correct observation. We now provide the complete anonymized Excel database used for the current research and all the statistical analyses.

Conclusions

Make sure to add a bullet point

Response: We thank the reviewer for this comment. We don’t know if a bullet point it is appropriate as for journal style. If so, we have provided a separate file with the bullet list.

Limitations

It is always interesting to point it out

Response: We thank the Reviewer for this comment. We added a Limitations section.

Reviewer #2: The authors attempted to assess the effects of the COVID-19 pandemic on research activities of a univeristy in Italy and identify the most critical issues, from a crosssectional study. The findings may provide some evidence of the pandemic impacts on the research community in an insitution.

The manuscript requires some substantial revisions before consideration of the journal:

1. the literature review was not organised and presented with sufficient evidence

Response: we thank this reviewer for the comment. We have largely integrated both introduction and discussion with the available pertinent literature. Please see red text.

2. the statistical analysis part was not elaborated and explained clealry, especially how the criteria and assumptions of a partciular statistical analysis were met, e.g. some questions in the survey allow selecting multiple answers, how did these considered in the statistical tests

R. We thank Reviewer #2 for his considerations and constructive criticism. Survey questions that allowed multiple answers were analysed and presented as follows: 1) in Table 2 with regards to the description of the research-related issues (no statistical testing); 2) in Tables 3 through 4 with regards to the overall research-related issues experienced based on academic position and according to the impact of gender, age, academic field (using ANOVAs, with no post-hoc testing); 3) in Tables 5 and 8 to assess independent predictors of the overall number of research related issues and the presence of economic burden (using multiple linear regression); 4) in Tables 6 and 7 to describe and assess the reasons for economic burden and suggestions to improve academic research, according to abovementioned subgroups (using ANOVAs, with no post-hoc testing).

Given the exploratory nature of our study, in which data are collected with an objective (*“to explore the impact of the COVID-19 pandemic on research activities and in the attempt to identify the most critical issues”*), rather than with prespecified key hypotheses, we chose not to adjust for multiple comparisons, according to relevant literature on the topic [(10.1530/EJE-20-1375,](https://doi.org/10.1530/EJE-20-1375) [10.1016/j.joca.2016.01.008,](https://doi.org/10.1016/j.joca.2016.01.008) [10.1016/S0895-4356(00)00314-0)](https://doi.org/10.1016/S0895-4356(00)00314-0). As such we place low emphasis on specific significant comparisons, but rather focus on the description of the research cohort and on exploring risk factors for greater research burden. Lastly, we employed bootstrapping on 2000 samples as a robust regression methods to account for non-normal data distribution, reporting bias-corrected accelerated (BCa) 95% confidence intervals and adjusted R^2^ values (according to Nagelkerke) to describe the fit of our models. We have made these points clearer to the reader in the manuscript.

3. the survey adopted was self-designed, some elaboration of the evidence of referencing to literature is required

Response: we thank the Reviewer for his/her correct observation. The questionnaire was indeed self-designed from the authors, who serve(d) as Governance Research Team at Sapienza University of Rome, based on the available literature on the topic, referenced in the Introduction and the Discussion sections. Specifically, the development of the survey focused on exploring the differential impact of the pandemic on different academic subgroups, expecting a greater toll on women (PMID 32409466), younger researchers and Ph.D. students (PMID 32493949**),** specific research activities (PMID 36530543), and researchers involved in clinical trials (35050229), etc. All these aspects are now better discussed in the Introduction section and the methodology is more clearly presented in Lines 149-151.

4. the discussion part appears lacking in-depth discussion, some substantiation of the literature is required

Response: we thank this reviewer for the comment. We have largely integrated both introduction and discussion with available literature. Please see red text.

5. an Italian academic institution was selected as the study site, given its specificity in its organization/system/roles of academic personnel as stated by the authors, the authors are required to add to explain how the findings would be useful to the wider academic community in other countries

Response: we thank this reviewer for the comment. As now clearly stated in the text, Sapienza is the largest university in Italy with many excellent departments. Academics of all degrees have taken part to this survey, therefore the authors think that results could be useful to a wider academic community. In order to meet this appropriate remark of the reviewer, we also proposed a modified title of the article which reflects the impact of pandemic of a very representative academic research community and on potential other institutions.
