# Supplementary material for: “The impact of the COVID-19 pandemic on research activities: A survey of the largest Italian academic community”
Source: PLoS One. 2024 Jun 25;19(6):e0304078. doi: 10.1371/journal.pone.0304078 (PMC11198756; doi:10.1371/journal.pone.0304078)
Supplement: Attachment — Submitted filename: Comments to the Author 2502.docx [file pone.0304078.s005.docx]

**Point-by-point Responses to Comments to the Authors**

Reviewer #1: Dear authors,

The manuscript is about an important topic and at several moments you addressed it well. However, I have a few suggestions that may improve your research report.

Study report and methods:
please check the two references bellow about survey report. They are about the checklists you can use to increase quality and transparency of your research report. I suggest to use it, add to your methods section and to the supplementary files the checklists done.

Eysenbach G. Improving the quality of Web surveys: the Checklist for Reporting Results of Internet E-Surveys 1. (CHERRIES). J Med Internet Res. 2004 Sep 29;6(3):e34. doi: 10.2196/jmir.6.3.e34. Erratum in: doi:10.2196/jmir.2042. PMID: 15471760; PMCID: PMC1550605.
2. Sharma A, Minh Duc NT, Luu Lam Thang T, Nam NH, Ng SJ, Abbas KS, Huy NT, Marušić A, Paul CL, Kwok J, Karbwang J, de Waure C, Drummond FJ, Kizawa Y, Taal E, Vermeulen J, Lee GHM, Gyedu A, To KG, Verra ML, Jacqz-Aigrain ÉM, Leclercq WKG, Salminen ST, Sherbourne CD, Mintzes B, Lozano S, Tran US, Matsui M, Karamouzian M. A Consensus-Based Checklist for Reporting of Survey Studies (CROSS). J Gen Intern Med. 2021 Oct;36(10):3179-3187. doi: 10.1007/s11606-021-06737-1. Epub 2021 Apr 22. PMID: 33886027; PMCID: PMC8481359.

*R. We thank the Reviewer for her detailed and constructive comments and the suggestion to include the use of a survey checklist. We adopted the CROSS checklist by Sharma A. et al. and we present it in Supplementary material as Appendix S2. We further describe its use in the Methods section [Lines 149-151].*

The authors approach several important topics such as gender inequalities and disparities through science. However, it is important to review some of the writing to avoid misleading conceptions. I suggest to improve the references used throughout the manuscript and to be careful when it comes to date of publications as well. Specially because early COVID-19 studies didn't have sufficient data.

*R. We thank the Reviewer for this comment. We have revised the whole manuscript with the support of an English mother-tongue person. In addition, we checked the references throughout the manuscript and we added new refs as suggested or appropriate*.

Introduction:
The authors already worked on the introduction, still there are still some things that should be taken into account.
Why the study is important? What it can add to the scientific knowledge? (observations at the file attached).

*We thank the reviewer for this comment. We have acknowledged all observations related to the Introduction in the manuscript. See text .*

Methods:
Please explain more about how the survey was developed. Use the checklists suggested above.
Regarding the survey - what the authors mean about “family organization”?
Regarding the “economic burden” - did you asked about it in the previous year? Since the beginning of the pandemic? Did you consider a burden as a reduction at family income or just the respondent income?

*R. We thank the Reviewer for her observations. For the use of checklists see the point mentioned above. The questionnaire proposed to the participants was referred to the period going from the onset of the pandemic, up to April 2021. As it can be seen from the online survey administered to all participants (provided in full in the Supplementary material), the terms “family organization” (to make it more clear we now define this field as “impact on family organization”), “economic burden”, etc. were indeed chosen by the participants in their responses. As such, no specific definition or interpretation can be given by the authors; this is what the respondents chose the answer(s) as they felt it appropriate on the basis of their subjective experience. We agree with the Reviewer that it would be interesting to have more insights on the impact of the pandemic on “family problems” or “economic burden”, but this should be addressed in specifically designed studies.*

Have you performed a pilot study to test the survey?

*R. We did not conduct a pilot study. This is an obvious consequence of the sudden onset of the pandemic which prevented any previous experience.*

Stats: please add the tests results as supplementary information. the results of the stats tests will assist others to better understand your results and will increase transparency.
Bootstrapping is not commonly used. Have you tested for normality before using bootstrapping? The test may result in a few limitations for stats inference that was not addressed by the authors on the limitation section.
More comments are at the file attached.

*R. We thank the Reviewer for her observations. We would like to observe that the complete dataset was already made publicly available using the data repository Figshare (see point above) (we checked it again and it is fully accessible) in the previous step of the revision process, and we fully describe in the Methods section the complete statistical analysis employed. Furthermore, the results are completely presented in Tables 1 through 8. As such we believe full transparency is guaranteed, both to the Editor, the Reviewers and the readers.*

*We indeed did analyze data distribution, and employed a robust and vastly used regression method, namely bootstrapping, to account for non-normal data, as specified in the Methods section:*

*“Data distribution was visually inspected by analyzing the respective histograms and normality plots. […] A robust approach using bootstrapping for 2000 samples was employed to account for non-normal data distribution, and bias-corrected accelerated (BCa) 95% confidence intervals were calculated and reported.”*

*Given the numerosity of the study sample size, we believe no specific limitations to study results and conclusions may arise from the use of bootstrapping.*

Results:

It would be interesting to add a flowchart of the survey production and results of respondents - at the beginning of the results section.
Please check the references I add previously about survey reports.

*R. We thank the Reviewer for her suggestion. As specified in the Methods section the online survey employed was collegially developed by the Sapienza University Governance Research Team, after careful review of the available literature on the topic, at the time of study design. Furthermore, considering that the study was cross-sectional in nature, and respondents were only asked to complete the survey at a single time-point, we believe that a flowchart would not add sensible information to the readers. With regard to online surveys checklist, we adopted the CROSS checklist by Sharma A. et al., as specified above.*

Discussion:
It requires a few adjustments to correlates the authors findings with the current literature. Use this section to add how your main findings can be useful for future policies and practices in science.
Please check the file attached with more observations.

*R. We thank the reviewer for this comment. We have put our results in the context of the existing available literature and added several new references. We introduced a new paragraph about the relevance of our work (lines …..).*

Conclusion:
What are the main findings of your study? And a final recommendation?
The conclusion is very broad and could reinforce the main findings and how it impacts science.

*R. We have added a short summary of our findings in the conclusion.*

References

Please make sure to cite the papers at the end of each sentence;
some references are probably in the wrong place - please check the file attached.
Add more relevant references to support you idea. I suggested a few that you are not obligated to use.

*R. We thank the reviewer for this comment. We have added new references and mentioned them at the end of each sentence in the text.*
