# Supplementary material for: “The impact of the COVID-19 pandemic on research activities: A survey of the largest Italian academic community”
Source: PLoS One. 2024 Jun 25;19(6):e0304078. doi: 10.1371/journal.pone.0304078 (PMC11198756; doi:10.1371/journal.pone.0304078)
Supplement: Attachment — Submitted filename: Reviewer.docx [file pone.0304078.s006.docx]

Reviewer #1: Dear authors,

there is just a few minor points to be revised. I appreciate all the comments and clarifications on the previous review.
Congratulations on your research report.

"All these factors may reduce opportunities for ERCs funding applications and for job applications, in the current context of job cuts and hiring freeze due to global financial pressures. On the other hand, many journals have granted deadline extensions for submitting academic work, financial reporting of ongoing projects has been postponed by some research institutions and universities, and the time limit for the expense of funding derived from grants has often been extended." - > please make sure to add the reference to support this information.

Thank you for your comment. We have added some references to the bibliography (n 38-39)

Please correct:

'Although out study does not reflect a multicenter approach, it has been conducted in the largest Italian University and one of the largest academic communities in Europe and its findings are largely consistent with previous similar studies."to (...) our study (...)

Thank you, we have corrected the typo.
